# Should pre–measurement physical activity be standardized in muscle thickness and stiffness evaluations? – A randomized controlled four arm cross-over study

**DOI:** 10.1186/s12880-026-02373-5

**Published:** 2026-04-29

**Authors:** Konstantin Warneke, Gerit Plöschberger, Manuel Oraze, Daniel Jochum, Stanislav D. Siegel

**Affiliations:** 1 Institute of Sustainability Psychology, Leuphana University Lüneburg, Lüneburg, Germany; 2 Institute of Human Movement Science, Sport and Health, University of Graz, Graz, Austria; 3 Viktor Frankl Hochschule, Pädagogische Hochschule Kärnten, Klagenfurt am Wörthersee, Austria; 4 Institute of Human Motion Science and Exercise Physiology, Friedrich Schiller University Jena, Jena, Germany

**Keywords:** Ultrasound, Muscle swelling, Acute muscle adaptation, Tissue stiffness, Myotonometry, Muscle property

## Abstract

**Background:**

High standardization is of crucial relevance for reliability in imaging diagnostics. When quantifying muscle properties (muscle thickness and stiffness) by ultrasound or myotonometry, internal validity can be compromised by examiner-related factors and participant biologic variability. A frequently neglected source of bias is pre-measurement activity, which may acutely alter muscle perfusion and muscle blood inflow.

**Methods:**

The acute influence of different physical activity routines on tissue parameters was investigated in 30 healthy participants (16 m, 14f). Ten minutes before, immediately before, immediately after and 10 min retention of cycling, jogging, calf raises or control, muscle thickness and stiffness measurements via shear wave elastography (SWE) and myotonometry were measured.

**Results:**

Reliability was excellent for muscle thickness (ICC = 0.94–1.00; CV = 1.7–9.1%), good-excellent for SWE stiffness (ICC = 0.68–0.97; CV = up to 26% for inter-day) and myotonometry (muscle ICC = 0.77–0.98; CV = 4.0–17% tendon 0.86–0.93 (CV = 11–17%). Muscle thickness significantly increased after calf raises (d = 1.60, 10.3%) and jogging (d = 0.60, 3.0%), without effects after cycling or control. Shear-wave elastography showed muscle stiffness decreased after calf raises (d=-0.73, -16.7%). Myotonometry indicated a stiffness increase (d = 1.04, 20.1%). The 10-minute retention showed consistent effects for muscle thickness (d = 0.80, 5.3%) and stiffness (SWE: d = 0.78, 21.1%, myotonometry: d=-0.82, -13.0%).

**Conclusion:**

Pre-measurement activity could systematically affect muscle thickness and stiffness with dependence on activity type and intensity. This highlights the importance of monitoring pre-measurement activity to minimize potential reliability issues as this, depending on several potential moderators, could enhance the random error if within sample pre-measurement activity is not standardized. Before ultrasound evaluation, for some activity (i.e. calf raises), > 10 min of rest was required to diminish this bias.

**Supplementary Information:**

The online version contains supplementary material available at 10.1186/s12880-026-02373-5.

## Background

In sports and medical settings, the evaluation of muscle properties such as muscle thickness and stiffness is of crucial importance. In research, scientists evaluate the effects of interventions on different muscle parameters such as muscle thickness. Hereby, increases of the muscle cross-sectional area mark hypertrophy after exercises [1], while decreases confirm muscular atrophy after disuse, immobilization [2, 3] or age-related sarcopenia [4, 5]. Additionally, a growing interest emerges in muscle stiffness investigations of the muscle and tendinous structures. While, for instance, stretch-shortening dominant activities such as the (drop) jumps or running benefit from higher (tendon) stiffness [6, 7], with age, muscle stiffness seems to increase, and therapeutical interventions oftentimes seek to reduce muscle stiffness parameters in older adults [8].

Especially in scientific settings, the precise and valid parameter determination becomes paramount to attribute measured changes to the intervention instead of measurement errors due to lack of standardization. The gold standard measurement for muscle size is magnetic resonance imaging. Testing seems unbiased from subjective influences and was highly reliable [9, 10]. Nevertheless, evaluation is cost- and time-consuming while being additionally locally bounded. This makes the evaluation unapplicable in several settings [10, 11]. To substitute, most scientists and clinicians use ultrasound devices to monitor muscle architecture. When using shear wave elastography (SWE), this procedure allows the determination of muscle stiffness in addition to muscle thickness or muscle cross-sectional area [12, 13]. However, therapeutic facilities commonly lack access to SWE and seek muscle mechanical property evaluations via myotonometry, which was outlined as a viable tool for clinicians [14, 15].

SWE as well as myotonometry were outlined to be sufficiently reliable with intraclass correlation coefficients (ICC) of 0.80–0.99 for ultrasound [16] and ICC = 0.72–0.99 for myotonometry [14, 16]. Generalized statements, however, reflect unawareness of the difference between device and protocol reliability, as the reliability of these investigations crucially depends on the evaluated sample and sufficiency of surrounding moderator control [17]. Accordingly, the outlined heterogeneity in ICCs might be attributed to meaningful measurement errors typically stemming from (a) lack of measurement standardization and/or (b) biological variability of participants [18–20], both resulting in a random error.

 For instance, in ultrasound measurements the investigator experience meaningfully influenced the precision (meaning random scattering [21]). Hereby, measurement error sources refer to inconsistencies in applied pressure, angle or rotation of the device [16, 21]. Effects were even larger for SWE, where only 1 N affected reliability [12]. These standardization problems, however, primarily refer to a limitation arising from the examiner.

Further sources of variance might stem from insufficient standardization of surrounding parameters affecting the participants. Ultrasound is commonly performed at the beginning of data collection sessions, as subsequently performed tests could bias validity of ultrasound evaluations. Maximal strength tests or training enhance the physical activity level, causing an increased muscle perfusion, which cause muscle swelling of up to 14.5% [22, 23]. Additionally, when exercise was exhausting, literature referred to inflammatory processes that could enhance intra- and extracellular fluids with meaningful effects on muscle structure and properties [24, 25]. Such effects could even increase muscle and connective tissue stiffness [26].

As a consequence, in most scientific literature, ultrasound evaluations were performed at the start of testing to minimize activity induced alterations to muscle thickness and stiffness assessment. It is, however, surprising that in muscle imaging protocol descriptions, standardizing pre-measurement activity level of participants and surrounding conditions are scarce. For instance, Ruple et al. [27] described that participants were shuttled to MRI scans with participants resting five minutes before measurement to allow body fluid stabilization. In some investigations, participants were asked to refrain from physical exercises 24 h prior to the actual investigation [28] but did not describe how participants arrived to the lab (e.g. by bike). Other works did not describe avoidance of pre-measurement activities at all but started ultrasound after 10 min of rest after arrival to the lab [29]. This heterogeneity might be problematic and could affect acquired muscle thickness and/or stiffness evaluations meaningfully. Imagine that some participants arrived at the test by bus, others by bike while others walked. From thousands of ultrasound measurements performed by our team, we hypothesized that these activities must be standardized and approaches (e.g. resting duration before actual measurement) to control such effects are needed. This is crucial to minimize participant related random errors in sensitive muscle mechanical evaluations.

To raise awareness for the relevance of pre-activity effects on muscle thickness and stiffness evaluation, this work systematically investigates the acute effects of different physical activity routines on ultrasound muscle thickness and SWE muscle stiffness evaluations in the plantar flexors, supplemented by myotonometry to enhance practical relevance for clinicians without access to SWE. Therefore, it was hypothesized that active conditions would (a) cause a significant acute increase in muscle thickness (muscle swelling) and (b) significantly affect stiffness parameters (systematic bias). Literature showed that acute muscle swelling diminishes quickly. Therefore, to evaluate whether a practically relevant ten-minute rest before muscle thickness evaluations start was sufficient [27, 29], a 10-minute retention test [30] will be performed.

## Methods

The study was designed as a randomized four arm cross-over study of acute exercise effects with 4 measurement time points per test session. Therefore, each participant underwent 4 × 4 muscle mechanical property (muscle stiffness and thickness) investigations via ultrasound and myotonometry. Tests were performed immediately after arrival (pre0), after a 10-minute resting period and before the intervention (pre10), after the intervention (post0) as well as after another 10-minute retention period (post10). All subjects participated in 4 conditions, including cycling, jogging, calf raises or a passive control condition on four separated days with at least 48 h in between to reduce potential influence of the previous test session.

### Participants

Sample size estimation was performed with G-Power using an effect size of 0.8 to detect large effect sizes [31], α-error = 0.05 and statistical power (β – 1 error) = 0.95 for four groups and four measurement time points results in an overall sample size of 24. To enlarge statistical power and account for potential dropouts, a sample size of 30 participants (age: 28.26±4.32 years, mass: 72.07±13.94 kg, height: 174.32±8.84 cm) resulted in effect size calculation from 120 data points. These were recruited from the university campus at the local university and consisted of 14 females (age: 24.13±3.76 years, mass: 62.34±6.64 kg, height: 163.31±5.87 cm) and 16 males (age: 25.52±5.02 years, mass: 76.65±7.72 kg, height 179.59±4.31 cm). Inclusion criteria comprised regular participation in structured sports- and exercise programs at least twice per week (e.g. in the gym, university sport program, structured team sports) and the ability to perform 10 min of jogging or cycling without exhaustion. This inclusion criterion was chosen to avoid that the following intervention induced different acute responses across the sample, as trained participants with regular physical activity might respond differently to activity compared to their untrained and sedentary counterparts [32, 33]. Furthermore, to avoid individual outliers and measurement complications in the muscle property investigations, overweight participants (BMI < 30) were excluded as the muscle tissue measurements (e.g. stiffness) could be biased by different inter-individual parameters [34], for instance, if too much fat tissue lies over the muscle or tendon. Furthermore, all participants had to be healthy, meaning the absence of chronic cardiovascular, neurological or orthopedic indications or acute infects, which could cause any problems when performing the 3 active interventions. Participants were excluded if they reported delayed onset muscle soreness (DOMS) from pre-measurement activity (e.g. own training) and were therefore instructed to avoid exhaustive exercise (e.g. heavy resistance training, unfamiliar training stimuli) 24 h before lab arrival [28]. The study was conducted in accordance with the declaration of Helsinki. All participants provided written informed consent. The study protocol was approved by the local university ethical review board (University of Graz ethical review board: GZ. 39/175/63 ex 2023/24).

### Randomization

All participants performed all conditions in a randomized order with a minimum rest between days of 24 h and a maximum of 72 h to ensure comparability between trials. Randomization was performed via lottery with allocation concealment. In detail, after the initial evaluation on the day (after pre0, before pre10) the participant picked one out of four paper sheets without seeing the number written on the paper, that codes the intervention. The *1* allocated the participant to the calf raise condition, *2* to the jogging condition, *3* to cycling and *4* coded the passive control condition. Participants were therefore blinded for the upcoming intervention until the intervention started.

### Muscle and tendon evaluation

Muscle evaluation was performed via ultrasound SWE (Aixplorer V12.3, Supersonic Imaging, Aixen-Provence, France) and myotonometry (Myoton AS, Tallinn, Estonia) always on the dominant leg (the leg with which participants would kick a ball [35]) while participants were lying prone on a massage table. Participants were instructed to relax on the physiotherapy table, and the following measurements were performed in a relaxed joint angle position. The stiffness in the Achilles tendon was only investigated with the MyotonPRO (Myoton AS, Tallin, Estonia), as the Aixplorer maximal resolution was insufficient to monitor maximal values.

#### Ultrasound elastography investigation

**Fig. 1:**
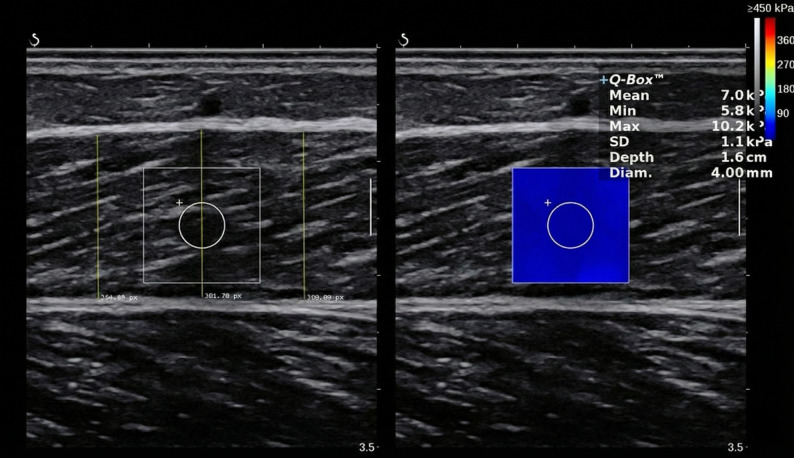
exemplifies the gastrocnemius medialis muscle thickness and shear wave elastography (SWE) evaluation. The vertical lines show three different measures of muscle thickness by determining the distance between the fascia layers. The left distance measures were standardized in the left third of the picture, the right distance in the right third of the figure and the midline measured the distance in the middle of the muscle, which was performed based on the standardization marks of the ultrasound evaluation software. The SWE region of interest positioning was pre-set by the device for maximal standardization

Ultrasound muscle architecture and viscoelasticity evaluation is one of the most frequently applied investigation technique across the literature with numerous studies showing sufficient reliability and validity [36]. For ultrasound muscle thickness and stiffness measurements, the participants were positioned in a prone position and the evaluation was conducted in the medial gastrocnemius head unilaterally [16]. In detail, the measurement position was marked in a standardized procedure. The distance between the proximal and distal muscle tendon junction was marked with a permanent, water resisting marker and measurement was conducted at 50–70% measured from distal landmark, depending on the individual anatomy of the participant. The upper and lower borders in which the ultrasound probe was placed was determined to re-trace the measurement spot, in case a participant lost the marks over the intervention period. In each of the four tests, the positioning was refreshed, and participants were introduced to re-mark every morning, especially after showering. Data were collected using a linear probe (SuperLinear 15 − 4, 4–15 MHz, Vermont, Tours, France) ultrasound device with SWE module turned on (Aixplorer V12.3, Supersonic Imaging, Aixen-Provence, France). Imaging was performed by placing the region of interest in the mid-point of the muscle with fascia superficialis and deeper fascia layer being parallel. Muscle thickness was determined via three length measurements on the left (in the left third of the image) and the right side (right third of the image), as well as in the middle of the picture (see Fig. 1). Distance measurement placement was standardized via software specific marks to ensure that the muscle thickness was always measured at the same image location. The mean of the three distances was processed for further statistical calculations. Muscle stiffness was evaluated via SWE. To calculate stiffness values, an ultrasound (acoustic) impulse produced a shear wave which, in turn, cause a compression. Velocity of this wave is measured vertically to the impulse. Assuming a proportional relationship between stiffness and shear wave velocity, faster shear waves indicate stiffer tissue. The result was read off the Q-Box from each image provided by the device. The Q-Box placement was pre-set from the SWE device to standardize the location across images for muscle stiffness evaluation and ensuring sufficient reliability. In general, SWE muscle stiffness evaluation has been reported to be reliable [37, 38]. Each measurement was performed twice to determine intra-session reliability. A second investigator read off the stiffness values and the muscle thickness evaluation was performed independently with assessor blinded for the condition. Ultrasound evaluation was performed by an experienced investigator, who showed reliable ultrasound results in numerous previous investigations [16].

#### Myotonometry

The mechanical properties of soft tissues were evaluated using the MyotonPRO (Myoton AS, Tallinn, Estonia), a handheld device designed to quantify viscoelastic characteristics through the tissue’s response to a brief mechanical impulse (0.42 N) excerpted from pre-load pressure (0.18 N). The participants position on the table was maintained with myotonometry and ultrasound measurements to avoid any interferences or position changes of the leg. Therefore, the participant stayed in a prone position. The device provides objective measurements of oscillation frequency (Hz), dynamic stiffness (N/m), logarithmic decrement (representing tissue elasticity), mechanical stress relaxation time (ms), and creep. All parameters were automatically computed by the MyotonPRO based on the damped natural oscillation response of the mechanical impulse [39]. Prior to measurements, participants were positioned in a prone position on a massage table, with feet hanging over the edge to minimize tension in the lower limbs. The upper body was fully relaxed, arms rested alongside the torso, and the head was turned to the right. Skin-friendly markers were used to mark the measurement point to maintain consistency across trials and marked according to the manufacturer’s guidelines to ensure reproducibility: For Achilles tendon measurements, a point on the tendon was marked between the two malleoli of the ankle. For the gastrocnemius medialis, the measurement point was positioned at the center of the ultrasound marking to ensure consistent probe placement. For each measurement, the MyotonPRO probe was applied perpendicular to the skin at the pre-marked site. Measurements were initiated only after ensuring stable, full contact between the probe and the tissue. Care was taken to minimize external interference, such as muscle contractions or shifts in body position, during the assessment. The data from the MyotonPRO (the five measured parameters that can be read from the MyotonPRO per measurement: F = [oscillation] frequency, S = stiffness, D = elasticity, R = relaxation, C = creep) were synthesized in an excel sheet by a separated investigator to blind the assessor from previous results. In the literature, the MyotonPRO parameters reached intra-rater reliability for all MyotonPRO parameters in the gastrocnemius medialis ranged from 0.78 to 0.99 [15].

#### Interventions

**Fig. 2:**
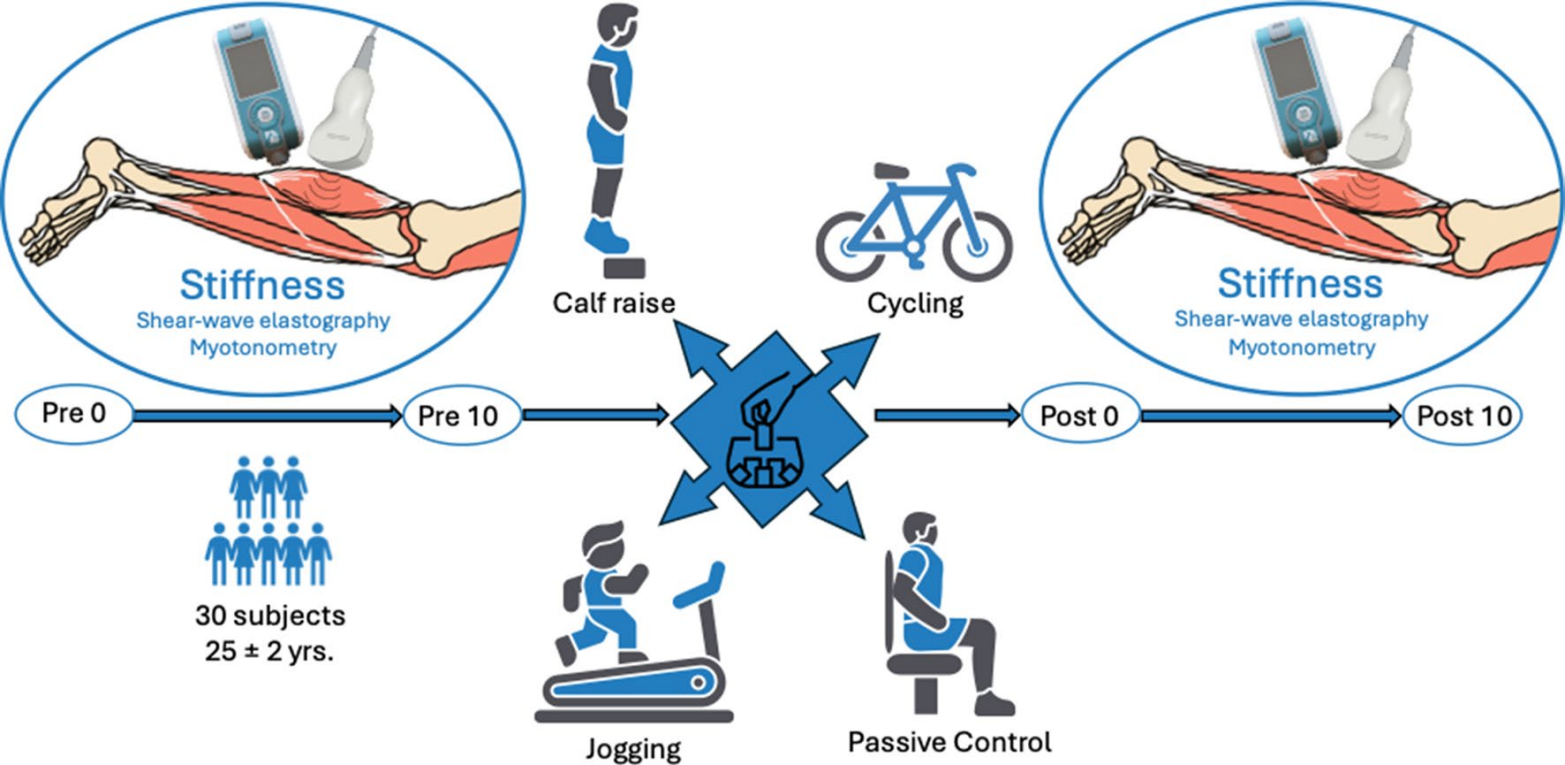
graphically illustrates the study protocol with condition allocation into one out of three interventions or the passive control condition

All participants adhered to the same protocol in principle, while conditions applied between the pre10 and post0 differed depending on the condition allocation. To systematically investigate acute activity effects of three different interventions a 10-minute rest before the pre-testing was ensured to counteract potential effects of different transportation modes between conditions and participants. However, to evaluate potential effects of unsystematic arrival activity on intra-day and inter-session reliability, the first evaluation of the participants muscle properties was performed directly after arrival. Afterwards, a 10-minute rest in a lying position was ensured before the pre10 test to (a) minimize unsystematic differences between the participants in the pre-test arising from prior activities and (b) evaluate intra-day reliability in the actual pre-test (pre10) session. After finalizing the test, all participants performed three active interventions and one passive control condition, to which they were allocated on a daily basis. These comprised (a) 5 × 12 repetition standing calf raises with weight added so that a maximum of 12 and a minimum of 10 repetitions were possible [22, 31], (b) 10 min of jogging with a perceived intensity of 12–14 on the BORG scale for moderate intensity on a running court [40], (c) 10 min ergometer cycling with an equivalent intensity at 60-70RPM [41] or (d) passive control, which included 10 min sitting on a chair. These interventions were chosen as potential and exemplary physical activities that could be performed prior to measurements, if surrounding activity was not standardized (e.g. some arrived by bike, bus or others could walk to the testing session or go by bus, and some others might go to the gym prior to arriving at the lab). After finalizing the intervention, the post0 was conducted. After the post-test, a follow up measurement was performed 10 min after the post-test to evaluate retention effects. The control condition consisted of 10 min resting on a chair passively. The study protocol is graphically illustrated in Fig. 2.

### Statistical processing

All analyses were conducted in RStudio (Posit Software, PBC, Boston, MA, USA). Raw Excel data (Version 16.100.2, Microsoft corporation, Redmond, WA, USA) were imported and reshaped to long format; two participants were excluded because they lacked data in ≥ 2 conditions. Results were reported as means (M) and standard deviations (SD). Reliability was evaluated intra-day (intra-session test-retest) and inter-day (Pre0, Pre10, Post0, Post10) for each outcome. Intra-day intraclass correlation coefficients (ICC) were computed with a two-way mixed-effects, absolute-agreement, single-measure model (ICC(3,1) [42, 43],

$$\:ICC\left(\mathrm{3,1}\right)=\frac{{MS}_{R}-{MS}_{E}}{{MS}_{R}+\left(k-1\right){MS}_{E}}$$

$\:{MS}_{R}$= mean square for rows.

$\:{MS}_{E}$= residual mean square.

*k* = number of raters per subject.

and inter-day ICC with two-way random effects, absolute agreement, multiple raters/measurements (ICC(2,k) using irr::icc [44].

$$\:ICC(2,k)=\frac{{MS}_{R}-{MS}_{E}}{{MS}_{R}+\frac{{MS}_{R-}{MS}_{E}}{n}}$$

$\:{MS}_{R}$= mean square for rows.

$\:{MS}_{E}$= residual mean square.

n = number of subjects.

From the ICCs the standard error of measurement (SEM)

$$\:SEM=SD\times\:\:\sqrt{1-ICC}$$

ICC = intra class correlation coefficient.

$\:{SD}_{baseline}$= standard deviation at baseline.

and the minimal detectable change at 95% confidence (MDC) were calculated.

$$\:MDC=z\times\:SEM\:\times\:\:\sqrt{2}$$

z = z-score for confidence level (e.g. 1.96 for 95% CI).

SEM = Standard error of measurement.

Agreement was further characterized by Bland-Altman analysis [45, 46] (mean bias and 95% limits of agreement) on within-pair differences. Additionally, the mean absolute error (MAE)

$$\:MAE=\frac{1}{n}\times\:\sum\limits_{i=1}^{n}\left|{x}_{i}-{y}_{i}\right|$$

*n* = number of data points.

*i* = index for each (paired) data point.

*x*_*i*_ = *i*-th data point in variable *x*.

*y*_*i*_ = *i*-th data point in variable *y*.

mean absolute percentage error (MAPE)

$$\:\mathrm{M}\mathrm{A}\mathrm{P}\mathrm{E}=\frac{1}{n}\times\:\sum\limits_{i=1}^{n}\left|\frac{{x}_{i}-{y}_{i}}{\frac{{x}_{i}+{y}_{i}}{2}}\right|*100$$

*n* = number of data points.

*i* = index for each (paired) data point.

*x*_*i*_ = *i*-th data point in variable *x*.

*y*_*i*_ = *i*-th data point in variable *y*.

and the coefficient of variation of the differences (CV) were calculated.

$$\:CV=100\times\:\frac{{SD}_{diff}}{M}$$

$\:{SD}_{diff}$= overall standard deviation of the difference between to repeated measurements.

$\:M$ = overall mean between to repeated measurements.

Systematic bias between paired measurements was tested with paired t-tests.

To assess experimental effects, a two-factor repeated-measures ANOVA with both factors within-subject using afex::aov_ez [47] (Type-III sums of squares): Condition (calf raises, jogging, bike, control) $\:\times\:$ Time (pre0, pre10, post0, post10) were conducted. Model assumptions were inspected via Q-Q plots for normality of residuals; given the robustness of RM-ANOVA, minor deviations from normality were tolerated. Sphericity was assessed via Mauchly’s test; when violated, Greenhouse-Geisser correction was applied. For consistency across outcomes and because several variables violated sphericity, the Greenhouse-Geisser-corrected degrees of freedom and p-values, were reported. For significant omnibus effects (α = 0.05, two-tailed), hypothesis-specific post hoc tests using estimated marginal means (emmeans) package [48]((i) within-condition changes over time, (ii) between-condition differences at pre10 and at post10, and (iii) difference-in-differences comparisons (e.g., [post0-pre10] control - [post0-pre10] calf raises) were evaluated. Multiplicity was controlled with Holm adjustment within each outcome and hypothesis family. For the ANOVA, partial eta squared (ηp²) was calculated as a measure of effect size and interpreted according to established guidelines (small: ηp²≤0.01, medium: ηp²≥0.06, large: ηp²>0.14). For the t-tests, Cohen’s *d* was computed to quantify effect sizes and interpreted following common conventions (small: *d* ≤ 0.20, medium: *d ≥* 0.50, large: *d* > 0.80) [49]. ICCs were interpreted following Koo & Li [42]: <0.50 = poor, 0.50–0.75 = moderate, 0.75–0.90 = good, and > 0.90 = excellent (models reported as ICC(A,1) or ICC(A, k) with 95% CIs).

## Results

### Intra-day reliability

Intra-day reliability for muscle thickness evaluation was classified excellent with ICC = 0.974–0.995 (0.963–0.996 95%CI). The random error quantification showed SEM = 0.231–0.498; MAE = 0.198–0.358 over the four testing days, corresponding to a CV = 1.716–3.744%; MAPE = 1.028–1.900%. No systematic measurement bias occurred within each test, see Table 1. Muscle stiffness evaluated via SWE showed good to excellent reliability with ICC = 0.881–0.972 (0.832–0.981 95% CI) with SEM = 0.424–0.803 and MAE = 0.449–0.575, corresponding to CV = 5.920–10.713%; MAPE = 4.697–5.822%. The MDC for SWE stiffness evaluation was 1.174–2.225 kPa, while no systematic bias was observed. Stiffness intra-day reliability via myotonometry ranged from moderate to excellent (ICC = 0.772–0.982, 0.709–0.990 95% CI), with random errors ranges for the SEM = 8.992–30.045 and the MAE = 9.884–12.384, corresponding to CV = 3.976–14.792%; MAPE = 3.608–4.666%. At pre10 and post0 there were significant systematic test-retest difference (*p* < 0.001), indicating systematically higher values were measured in the first test. These systematic test-retest differences with − 5.80 N/m and − 5.21 N/m were below the MDCs of 30.27 N/m and 24.93 N/m. Achilles tendon stiffness was evaluated via myotonometry, showing similar reliability as muscle stiffness (ICC = 0.788–0.927, SEM = 23.506–38.425 N/m; MAE = 23.366–27.438 N/m and CV = 4.136–6.728%; MAPE = 2.869–3.605%, without a systematic bias). The remaining myotonometry parameters are provided in Table S1.

**Table 1:** Test-retest reliability within each test over the evaluation session

Parameter	ICC (CI95)	SEM	MDC	CV (in %)	MAE	MAPE (in %)	Bias	Bias_p	LoA_lower	LoA_upper
Muscle thickness (in mm) pre0	0.974 (0.963–0.982)	0.498	1.379	3.744	0.265	1.436	-0.027	0.683	-1.415	1.360
Muscle thickness (in mm) pre10	0.981 (0.972–0.987)	0.425	1.177	3.172	0.358	1.900	0.086	0.130	-1.087	1.259
Muscle thickness (in mm) post0	0.991 (0.987–0.994)	0.310	0.859	2.257	0.266	1.436	-0.025	0.545	-0.889	0.838
Muscle thickness (in mm) post10	0.995 (0.992–0.996)	0.231	0.640	1.716	0.198	1.028	-0.001	0.962	-0.646	0.643
MyotonPRO Stiffness (in N/m) pre0	0.866 (0.811–0.906)	18.170	50.364	9.252	10.275	4.666	-0.618	0.801	-51.278	50.041
MyotonPRO Stiffness (in N/m) pre10	0.940 (0.898–0.962)	10.921	30.272	5.258	9.884	3.608	-5.795	0.000	-34.116	22.527
MyotonPRO Stiffness (in N/m) post0	0.982 (0.967–0.990)	8.992	24.925	3.976	10.339	3.616	-5.214	0.000	-28.130	17.701
MyotonPRO Stiffness (in N/m) post10	0.772 (0.685–0.837)	30.045	83.279	14.792	12.384	3.791	1.759	0.664	-81.933	85.451
MyotonPRO Stiffness AT (in N/m) pre0	0.788 (0.706–0.849)	38.425	106.509	6.722	27.438	3.581	1.973	0.703	-105.090	109.036
MyotonPRO Stiffness AT (in N/m) pre10	0.919 (0.884–0.943)	23.506	65.154	4.136	23.366	2.869	2.438	0.441	-62.964	67.839
MyotonPRO Stiffness AT (in N/m) post0	0.927 (0.896–0.949)	24.033	66.615	4.225	24.304	3.016	3.411	0.291	-63.308	70.129
MyotonPRO Stiffness AT (in N/m) post10	0.811 (0.737–0.866)	38.208	105.908	6.728	26.295	3.605	-5.098	0.321	-111.202	101.005
SWE Stiffness (in kPa) pre0	0.881 (0.832–0.916)	0.803	2.225	10.713	0.575	5.672	0.111	0.305	-2.118	2.339
SWE Stiffness (in kPa) pre10	0.945 (0.922–0.962)	0.541	1.500	7.169	0.556	5.394	-0.035	0.633	-1.543	1.473
SWE Stiffness (in kPa) post0	0.972 (0.959–0.981)	0.424	1.174	5.920	0.449	4.697	-0.071	0.215	-1.245	1.104
SWE Stiffness (in kPa) post10	0.963 (0.946–0.974)	0.486	1.348	6.834	0.546	5.822	-0.034	0.605	-1.389	1.322

Legend: ICC=Intraclass correlation coefficient, CI95 = 95% confidence interval, SEM=standard error of measurement, MDC = minimal detectable change, CV=variability coefficient, MAE=mean absolute error, MAPE=mean absolute percentage error, Bias=test-retest difference in measurement unit, Bias p = p-value of the paired sampled t-test, LoA=limits of agreement, AT=Achilles tendon, SWE=shear wave elastography

### Inter-day reliability

As for inter-day reliability, muscle thickness evaluation revealed good to excellent reliability with ICC = 0.939–0.976, (0.856–0.988 95%CI), with a SEM = 0.460–0.740 mm; MDC = 1.283–2.050 mm and a CV = 6.94–9.070% over all four testing and all measurement time points. SWE muscle stiffness inter-day reliability was poor to moderate (ICC = 0.679–0.786, 0.412–0.893 95%CI) with SEM = 0.910–1.000 kPa and MDC = 2.511–2.769 kPa, the CV was at 23.49–26.25%. Stiffness evaluation via myotonometry showed moderate to excellent reliability with ICC = 0.864–0.926 (0.621–0.962 95%CI) for muscle, as well as Achilles tendon stiffness (ICC = 0.817–0.861, 0.676–0.930). The SEM for muscle stiffness is reported with 11.010–20.950 N/m and a CV of 11.32–17.24% for muscle and SEM = 25.320–29.010 N/m and CV = 8.91–10.21% for Achilles tendon stiffness, respectively (Table 2). For further detailed information please see Table S2.

**Table 2:** Interday test-retest reliability for all testing conditions at the baseline and pre-test

Parameter	ICC (CI95)	SEM	MDC	CV (in %)
Muscle thickness (in mm) pre0	0.975 (0.956–0.987)	0.480	0.132	7.130
Muscle thickness (in mm) pre10	0.976 (0.957–0.988)	0.460	1.283	6.940
Muscle thickness (in mm) post0	0.939 (0.856–0.973)	0.740	2.050	9.070
Muscle thickness (in mm) post10	0.971 (0.946–0.985)	0.530	1.478	7.360
MyotonPRO Stiffness (in N/m) pre0	0.926 (0.868–0.962)	11.950	33.113	12.060
MyotonPRO Stiffness (in N/m) pre10	0.924 (0.865–0.961)	11.010	30.517	11.320
MytonPRO Stiffness (in N/m) post0	0.864 (0.621–0.944)	20.950	58.059	17.240
MytonPRO Stiffness (in N/m) post10	0.892 (0.795–0.947)	17.050	47.274	15.600
MyotonPRO Stiffness AT (in N/m) pre0	0.846 (0.728–0.922)	25.820	71.565	8.990
MyotonPRO Stiffness AT (in N/m) pre10	0.865 (0.761–0.931)	25.320	70.173	8.910
MyotonPRO Stiffness AT (in N/m) post0	0.861 (0.747–0.930)	27.600	76.513	9.730
MyotonPRO Stiffness AT (in N/m) post10	0.817 (0.676–0.906)	29.010	80.413	10.210
Stiffness (in kPa) pre0	0.682 (0.430–0.839)	0.920	2.544	24.380
Stiffness (in kPa) pre10	0.707 (0.475–0.851)	0.910	2.524	23.960
Stiffness (in kPa) post0	0.786 (0.601–0.893)	0.910	2.511	23.490
Stiffness (in kPa) post10	0.679 (0.412–0.839)	1.000	2.769	26.250

Legend: ICC = Intraclass correlation coefficient, CI95 = 95% confidence interval, SEM=standard error of measurement, MDC = minimal detectable change, CV=variability coefficient, AT=Achilles tendon, SWE=shear wave elastography

### Within-condition changes (pre0 to pre10) and between conditions at pre10

A systematic within-condition effect from pre0 to pre10 was detected for muscle stiffness in the control group when assessed by myotonometry (*p* = 0.002). In contrast, no within-condition effects were observed for Achilles tendon stiffness, for muscle stiffness measured by elastography, or for muscle thickness across any groups (all *p* = 0.052–1.000). Nevertheless, small but statistically significant shifts were present in the control condition for specific myotonometry-derived parameters, as detailed in Table S5. Furthermore, there were no significant between conditions effects at pre10 (*p* = 0.179–1.00) (see Supplemental Material).

### Acute changes in muscle thickness and stiffness

#### ANOVA overview

Across all muscle parameters, two-way ANOVA showed significant Time effects, as well as Time$\:\times\:$Condition interaction (all *p* < 0.05) with moderate-to-large effect sizes (η_p_²=0.086–0.520; see Table 3). Table S4 shows the results of the other variables.

**Table 3:** Test descriptives separated for the intervention conditions with the following two-way ANOVA results for muscle parameter

	Condition	Pre0	Pre10	Post0	Post10	Main Effect Condition	Main Effect Time	Time$\:\times\:$Condition Interaction
Muscle Thickness (mm)	Calf Raise	18.72±3.06	18.60±2.98	20.72±3.39	19.70±3.32	F(2.53, 68.20) = 4.68 *p* = 0.008 ηp² = 0.148	F(2.22, 60.02) = 17.51 p = < 0.001 ηp² = 0.393	F(5.49, 148.23) = 12.67 p = < 0.001 ηp² = 0.319
	Jogging	19.16±3.08	19.18±3.20	19.78±3.09	19.37±3.21			
	Cycling	18.95±3.17	18.89±3.06	18.98±2.99	19.04±3.03			
	Control	19.02±3.17	18.98±3.06	18.81±3.08	18.77±3.17			
Stiffness (kPa)	Calf Raise	10.66±2.19	10.51±2.64	9.04±2.13	8.73±2.14	F(2.21, 59.70) = 3.19 *p* = 0.043 ηp²=0.106	F(2.42, 65.29) = 7.32 p = < 0.001 ηp² = 0.213	F(6.23, 168.16) = 5.13 p = < 0.001 ηp² = 0.160
	Jogging	10.80±2.50	10.90±1.85	9.98±2.69	10.21±2.54			
	Cycling	10.32±2.36	10.79±2.34	10.29±2.24	10.43±2.36			
	Control	10.51±2.64	10.71±2.21	11.18±2.57	11.25±2.30			
MyotonPRO Stiffness (N/m)	Calf Raise	279.75±40.73	273.63±37.42	346.93±91.33	317.02±70.95	F(2.31, 62.45) = 10.10 p = < 0.001 ηp² = 0.272	F(2.04, 55.00) = 12.97 p = < 0.001 ηp² = 0.324	F(2.85, 77.03) = 15.02 p = < 0.001 ηp² = 0.357
	Jogging	281.50±50.09	277.27±46.45	290.86±58.46	286.21±51.38			
	Cycling	273.71±46.41	273.16±34.68	270.38±38.92	282.10±58.59			
	Control	288.52±54.38	279.67±54.38	278.82±51.03	277.30±49.57			

#### Within-condition changes (pre10 to post0/post10) in muscle thickness

**Fig. 3:**
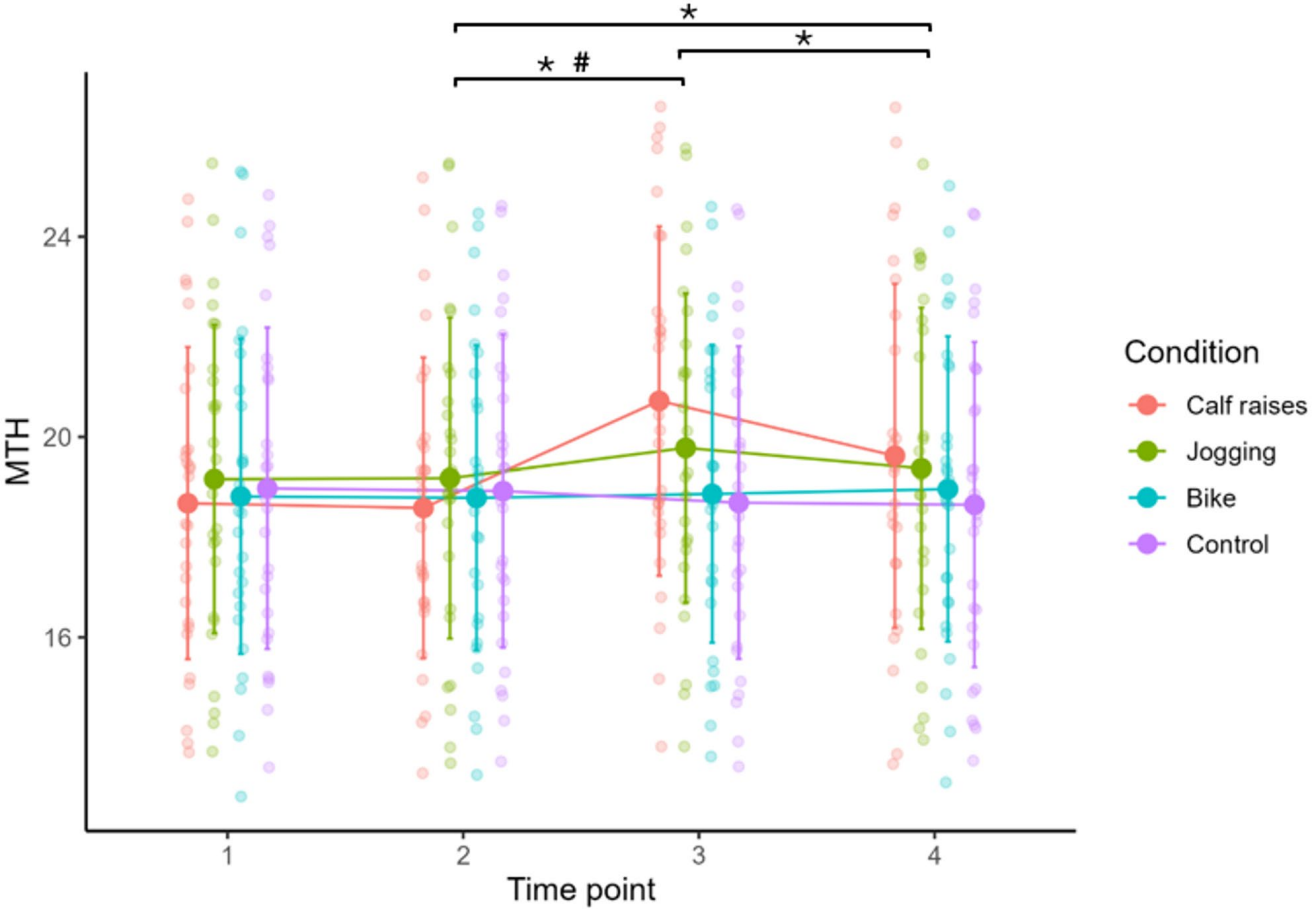
Mean and standard deviation of the muscle thickness (in mm) at different time points for all conditions, MTH = muscle thickness, Time point 1 = pre0 testing before 10 min of rest, Time point 2 = pre10 testing serving as the baseline value for the different interventions, Time point 3 = post0 testing as the post intervention test and Time point 4 = post10 reflecting the 10 min retention test. * Significant time effect for calf raises (*p* < 0.05). **#** Significant time effect for jogging (*p* < 0.05)

Significant pre10 to post0 and/or pre10 to post10 changes emerged for calf raise and jogging (see Table 4). Muscle thickness significantly increased in the calf raise condition (difference = 2.134 mm (10.3%), *p* < 0.001, d = 1.595) and remained elevated from pre10 to post10 (retention) (difference = 1.038 mm (5.3%), *p* = 0.001, d = 0.804) with a significant reduction between post0 and post10 (difference=-1.096 mm (-5.6%), *p* < 0.001, d=-0.957). Another significant increase was observed in the jogging condition from pre10 to post0 (difference = 0.601 mm (3.0%), *p* = 0.022, d = 0.602), without a significant retention effect. Cycling as well as the control condition showed no significant changes over time in muscle thickness of the calf muscle (see Fig. 3).

**Table 4:** Significant within-condition changes between the Pre-Test, Post-Test and Retention-Test

Parameter	Condition	Time	Estimate	CI95	SE	t	df	*p*-value	Cohens d
Muscle thickness (mm)	Calf raises	pre10 – post0	-2.134	[-2.854, -1.414]	0.253	-8.442	27	< 0.001	-1.595
	Calf raises	post0 - post10	1.096	[0.480, 1.713]	0.217	5.063	27	< 0.001	0.957
	Calf raises	pre10 - post10	-1.038	[-1.732, -0.343]	0.244	-4.254	27	0.001	-0.804
	Jogging	pre10 - post0	-0.601	[-1.138, -0.064]	0.189	-3.184	27	0.022	-0.602
MyotonPRO Stiffness (N/m)	Calf raises	pre10 - post0	-68.250	[-103.527, -32.973]	12.391	-5.508	27	< 0.001	-1.041
	Calf raises	post0 - post10	27.732	[10.657, 44.807]	5.998	4.624	27	< 0.001	0.874
	Calf raises	pre10 - post10	-40.518	[-67.116, -13.920]	9.343	-4.337	27	0.001	-0.820
SWE Muscle Stiffness (N/m)	Calf raises	pre10 - post10	1.841	[0.573, 3.110]	0.446	4.133	27	0.002	0.781
	Calf raises	pre10 - post0	1.516	[0.400, 2.632]	0.392	3.869	27	0.002	0.731

Legend: CI95 = 95% confidence interval, SE= standard error, SWE = shear wave elastography, AT=achilles tendon, S= stiffness, t = t-value, df = degrees of freedom

For each parameter and condition, entries are paired-samples mean differences for Pre-Post (pre10-post0), Pre-Retention (pre10-post10), and Post-Retention (post0-post10)

#### Within-condition changes (pre10 to post0/post10) in stiffness via shear wave elastography

**Fig. 4:**
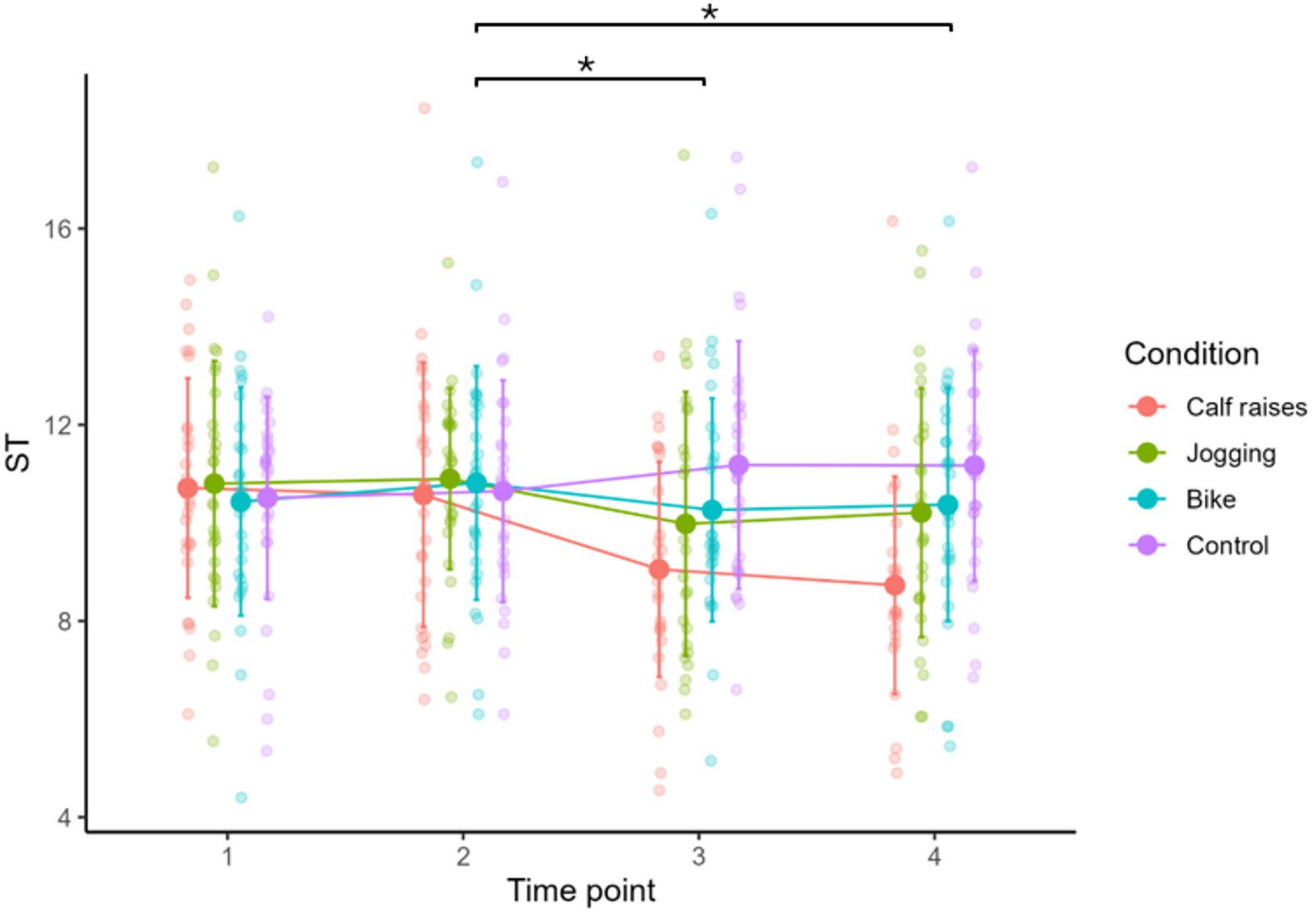
Mean and standard deviation of the muscle stiffness (in kPa) measured by shear wave elastography at different time points for all conditions, ST = muscle stiffness, Time point 1 = pre0 testing before 10 min of rest, Time point 2 = pre10 testing serving as the baseline value for the different interventions, Time point 3 = post0 testing as the post intervention test and Time point 4 = post10 reflecting the 10 min retention test. * Significant time effect for calf raises (*p* < 0.05)

Shear-wave muscle stiffness decreased significantly only in the calf raise condition: pre10 to post0 (difference=-1.516 kPa (-16.7%), *p* = 0.002, d=-0.731) indicated an exercise induced stiffness reduction that remained at post 10 (difference=-1.841 kPa (-21.1%), *p* = 0.002, d=-0.781 from pre10 to post 10). There were no significant effects associated with the jogging, cycling, and control conditions for muscle stiffness evaluated via SWE (all *p* > 0.05, see Fig. 4). Table S6 shows all effects for the other myotonometry variables.

#### Within-condition changes (pre10 to post0/post10) in myotonometry

In contrast to the stiffness results measured via SWE, the myotonometry indicated a significant stiffness increase from pre10 to post0 (difference = 68.250 N/m (20.1%), *p* < 0.001, d = 1.041) with a retention from post0 to post10 (difference=-27.732 N/m (-8.9%), *p* < 0.001, d=-0.874) for the calf raise condition. Nevertheless, no complete retention was measured at post10 (stiffness: difference = 40.518 N/m (13.0%), *p* = 0.001, d = 0.820) in the calf raise condition. Table S7 shows all significant effects for the other myotonometry variables. For the other conditions, effects were not significant (*p* = 0.203–1.000).

#### Between-condition contrasts of the pre to post change in muscle thickness (post-hoc analysis)

As indicated by the time effects, the post hoc analysis confirmed largest effects on muscle thickness and stiffness in the calf raise condition. Significant effects were reported compared to cycling, control, and jogging (difference=-2.367 mm to -1.533 mm, all *p* ≤ 0.001, d=-1.476 to -0.952). The percentage differences in post0 between calf raises and cycling, control and jogging are 9.8%, 10.9%, and 4.8%, respectively. Also, the jogging condition significantly increased muscle thickness compared to the control condition (difference=-0.834 mm, *p* = 0.043, d=-0.662) with 5.8% difference in post0. Post-hoc testing found no significant effect on muscle thickness for the cycling condition compared to the control (see Table 5).

**Table 5:** Significant pairwise between-condition contrasts of the within-subject change from Pretest (pre10) to Posttest (post0)

Parameter	Time	Condition	Rise	CI95	SE	t	df	*p*-value	Cohens d
Muscle thickness (mm)	pre10 - post0	Calf raises - Bike	-2.055	[-3.265, -0.844]	0.340	-6.048	27	< 0.001	-1.143
	pre10 - post0	Calf raises - Control	-2.367	[-3.447, -1.287]	0.303	-7.810	27	< 0.001	-1.476
	pre10 - post0	Calf raises - Jogging	-1.533	[-2.618, -0.449]	0.304	-5.039	27	0.001	-0.952
	pre10 - post0	Jogging - Control	-0.834	[-1.682, 0.014]	0.238	-3.502	27	0.043	-0.662
MyotonPRO Stiffness (N/m)	pre10 - post0	Calf raises - Bike	-72.232	[-116.990, -27.475]	12.561	-5.750	27	< 0.001	-1.087
	pre10 - post0	Calf raises - Control	-69.161	[-115.359, -22.963]	12.966	-5.334	27	< 0.001	-1.008
	pre10 - post0	Calf raises - Jogging	-54.661	[-90.976, -18.345]	10.192	-5.363	27	< 0.001	-1.013
SWE Muscle Stiffness (N/m)	pre10 - post0	Calf raises - Control	2.05	[0.093, 4.007]	0.549	3.732	27	0.030	0.705

Legend: SWE= shear wave elastography, CI95 = 95% confidence interval, SE= standard error, t = t-value, df = degrees of freedom

Entries show the estimated mean difference of the change (pre10-post0) between the two conditions (“first - second”). Negative values therefore indicate a larger increase from pre10 to post0 in the first condition compared with the second

#### Between-condition contrasts of the pre to post change in shear wave elastography

The only significant pre10 to post0 change for muscle stiffness measured by SWE that differed significantly was found for calf raises against the control (difference = 2.050 kPa, *p* = 0.030, d = 0.705) with − 19.0% difference between conditions in post0. No significant effects were found between jogging and cycling, jogging and control, cycling and control or calf raises versus cycling or jogging control. For graphical illustration, please see Fig. 4.

#### Between-condition contrasts of the pre to post change in myotonometry

While SWE measurement indicated a stiffness decrease in the calf raise condition, the direct opposite was found for stiffness when measured via myotonometry. The calf raise condition indicated a significant increase with difference=-72.232 to -54.661 N/m, all *p*<0.001, d=-1.087 to -1.013 compared to control, cycling and jogging. The percentage differences in post0 between calf raises and cycling, control and jogging are 26.4%, 22.6%, and 16.8%, respectively. The remaining graphics are provided in the Supplemental Material in the Figures S5 – S8).

### Acute changes in tendon stiffness

#### ANOVA overview

For Achilles tendon variables, significant condition effects (*p* < 0.05, ηp²=0.085–0.134) for all variables except logarithmic decrement (*p* = 0.091) were observed. No interaction effects were found in the Achilles tendon (see Table S3, Figure S1 – Figure S4 and excel table in the supplemental material).

#### Between-condition differences at retention (post10)

After 10 min of rest following the post0 test, muscle thickness in the calf-raise and jogging conditions remained significantly higher than in the control condition (calf raise-control: difference = 0.973 mm, *p* = 0.009, Cohen’s d = 0.656; jogging-control: difference = 0.724 mm, *p* = 0.007, d = 0.691) with 5.2% and 3.9% difference, respectively. At the same time point, muscle stiffness measured via SWE was significantly lower in the calf-raise condition compared with all other conditions (difference=-2.441 kPa to -1.480 kPa, *p* = 0.017 to < 0.001, d = 0.530–1.095) with 14.5–21.9% difference. Myotonometry indicated significantly higher stiffness after calf raises compared to other conditions (difference = 25.661–36.161 N/m, *p* = 0.027 to < 0.001, d = 0.553–0.903) with 9.0–13.1% difference and after jogging (difference = 10.500 N/m, *p* = 0.035, d = 0.512) compared with the control condition with 3.8% difference. The remaining effects in supporting parameters can be reviewed in the supplemental material (see Table S8 and excel table). 

## Discussion

The study aimed to investigate whether different exercise types cause acute muscle swelling and stiffness, the duration of these changes, and whether such effects should be considered when standardizing muscle property assessments using ultrasound SWE or myotonometry. Intra-session test-retest comparisons supports sufficient ultrasound investigation reliability with ICC = 0.97–0.99 for muscle thickness and ICC = 0.88–0.92 for SWE, with a random error quantification ranging between SEM = 0.23–0.50 mm, MAPE = 1.03–1.90% and SEM = 0.42–0.80 kPa, MAPE = 4.70–5.82% for muscle thickness and SWE, respectively. Therefore, within the sessions, high precision of the measurement was confirmed by all test-retest differences stayed far below the MDCs (0.64–1.38 mm for muscle thickness and 1.17–2.23 kPa for muscle stiffness). Similarly, MyotonPRO stiffness measurements reached moderate to excellent ICCs = 0.77–0.98 with comparable precision (MAPE = 2.87–4.67%) as reported for SWE. None of the mean bias-values reached the MDC (24.93–106.51 N/m). Between-conditions at pre10 (inter-day) were in accordance with the literature [15, 16, 36, 44] and classified as good to excellent for muscle thickness (ICC = 0.939–0.976, SEM = 0.46–0.74 mm, MDC = 0.13–2.05 mm) and moderate to good for the SWE stiffness measurement (intra- and inter-day reliability ICC = 0.679–0.786, SEM = 0.91–1.00 kPa, MDC = 2.52–2.77 kPa). For more details on precondition reliability evaluation see Tables 1 and 2 in the results section. Irrespective of the exact parameter, intra-day reliability metrics were consistently, albeit only marginally, higher than inter-day reliability across all modalities indicating that repeated measurements within the same day were more stable than those taken across days for SWE. Within a day, post-intervention measurements tended to show slightly higher ICCs and lower random error in a parameter-specific manner (muscle thickness: post10; MyotonPRO muscle stiffness: post0; Achilles tendon stiffness: pre10; SWE stiffness: post0), suggesting that a standardized intra-session warm-up potentially influenced measurement reliability, while the systematic error shows no clear systematic fluctuations. In contrast, our inter-day analyses showed more favorable ICC and SEM values prior to warm-up protocol compared to after it. This indicates that day-to-day variability in preconditioning and in the response to the warm-up might have increased random error and reduces ICCs, while the clinical relevance remains speculative and calls for further investigations [50, 51].

The pre10 test was the actual baseline test to answer the research question. The test served as the starting point for the four applied conditions as well as for 10-minute resting period. For muscle thickness, the results showed a significant muscle thickness increase in the plantar flexors after the 5 × 12 repetition standing calf raises, as well as after the jogging condition. In contrast, cycling and the control condition showed no significant changes from pre10 to post0. The 10-minute rest after the interventions was not sufficient for a full retention to pre10 baseline values (post10 condition effects showed significantly increased muscle thickness). In general, stiffness results were conflicting, depending on the measurement device. While SWE indicated a significant acute decrease in muscle stiffness without any retention, the myotonometry muscle mechanical property evaluation suggested even a stiffness increase. The latter did not show any changes for the Achilles tendon (*p* = 0.108–0.287).

### Acute muscle swelling and muscle thickness evaluations

Extensive research has examined the long-term effects of exercise on muscle structural parameters. Although acute effects have been studied [22, 24, 25, 31, 52], immediate changes in muscle properties (including muscle thickness and stiffness) remain generally less investigated [22, 24, 31]. Existing evidence indicates that different exercise modalities can induce acute increases in muscle thickness, reflecting muscle swelling. For instance, Kassiano et al. [22] reported swelling of the medial (8.8%) and lateral gastrocnemius (14.5%) immediately after four sets of 20 calf raises performed to failure in 17 young women. Similarly, Lesinski et al. [31] found a 5.7% increase in muscle thickness (d = 3.88) following 3 × 12 full range of motion (ROM) calf raises in 16 healthy participants; the effects remained significant after 15 min post-exercise (5.6%, d = 1.73). Comparable findings have been reported for the quadriceps: Csapo and colleagues [52] observed muscle swelling following 45° leg press training to failure in 41 participants, with elevated thickness persisting about 30 min. Taniguchi [25] confirmed similar effects after exhaustive leg extensions with 18 participants. Retention to baseline lasted about 30 min [52]. Similar effects were also observed for the biceps brachii [53, 54] with acute muscle thickness increases of up to 18% [54]. Yasuda et al. [55] reported even larger increases (20%) after combined blood flow restriction and low-intensity resistance training (20% of the 1 repetition maximum (RM)) to failure; significant differences to baseline were still present after 60 min. Similarly, Hill et al. [56] observed a significant muscle thickness increase after low load resistance training in the biceps and triceps after 75 repetitions of 30% maximal peak torque forearm curls (2.13±0.39 cm and 1.88±0.40 cm to 2.58±0.49 cm and 2.17±0.43 cm, respectively). Effects were accompanied by a significant increase in muscle blood flow in the working muscles. Partial retention of blood flow at five minutes posttest could explain the incomplete retention in muscle thickness. Notably, acute muscle swelling does not occur exclusively after resistance training. Brancaccio et al. [24] observed significant muscle thickness increases in the quadriceps femoris after an incremental cycling protocol to exhaustion in 35 men (32.1±3.3 mm to 34.9±3.0 mm).

Mechanisms refer to (a) inflammatory-induced oedema [57] (b) with enhanced extra- and intracellular fluid [25, 58] or (c) vascular perfusion and enhanced blood inflow to the working muscles [52, 54, 56]. Exhaustive exercise - especially when unfamiliar - can cause muscle microtraumatization through mechanical overload, also known as exercise-induced muscle damage (EIMD) or DOMS. This phenomenon is typically accompanied by an immediate enhancement of intra- and extracellular fluids and subsequent increases in inflammatory markers, such as IL-6, C-reactive protein, leukocytes, and/or creatine kinase [59, 60]. Several indicators argue against inflammation as the main explanation for immediate swelling. Firstly, even light exercise with negligible mechanical overload can cause an acute increase in muscle thickness [24, 55]. Secondly, inflammatory markers generally peak at 48 h post-stress, not immediately [61–63]. Supporting this, Taniguchi et al. [25] described that muscle thickness increased immediately after exercise, returned to baselines within about 1 h and increases again several after days. This description indicates two distinct mechanisms: While muscle swelling after days could refer to DOMS/EIMD induced inflammation caused edema, the presented findings can be explained via immediately enhanced blood flow (muscle vasodilation) and cardiovascular perfusion as a response to enhanced oxygen demands under physical activity [52, 64].

In line with previous research, the present study observed acute muscle swelling after light jogging, consistent with perfusion-related mechanisms. However, no earlier study explored such effects after light jogging. In contrast to Brancaccio et al. [24], the presented results did not confirm cycling induced muscle size changes. This discrepancy is probably attributable to the muscle involvement: the previous study measured the quadriceps femoris [24] – a muscle group directly involved in cycling – while the presented results refer to plantar flexor thickness – a muscle group that plays only a minor role in ergometer cycling when compared to calf raises and jogging (both target the plantar flexors).

### Acute changes in muscle stiffness

Acute adaptations in muscle stiffness are well documented in various research fields. Stretching [65, 66] and foam rolling [67, 68] effects were commonly evaluated using SWE and myotonometry to explain increased variance of flexibility via stiffness adaptations. While acute flexibility increases seem to originate at least in parts from stiffness reductions, the underlying mechanisms of muscle stiffness as a response to exercise are less discussed in the literature. When muscles contract, exothermal reactions are responsible for actin-myosin cross-bridging, causing an increased muscle temperature – potentially favorable for viscoelastic properties (i.e., decreased tissue viscosity = less movement resistance) [69, 70]. This mechanism might not exclusively improve contractile properties (which were not measured in the present study) but also cause a reduced passive tissue stiffness. In their meta-analysis, Warneke et al. [70] compared stretching and foam rolling to any concurrent alternative reported in warm-up research. They found no differences in ROM or stiffness between the activities, suggesting that general physical activity enhances muscle temperature and improves viscoelasticity. However, stiffness reductions are not limited to low-intensity / low-volume warm-up routines: Andonian et al. [71] observed quadriceps stiffness reductions in 50 extreme mountain marathon runners during and after their run, attributed to overuse or supraphysiological stress. Also, Sadeghi et al. [72] reported decreased stiffness parameters in most of the evaluated muscles 24 h after a long-distance run. However, it must be noted that this test was performed 24 h after running, limiting the comparability with the present results. Cycling also caused a significant decrease in muscle stiffness, with Morales-Artacho et al. [41] reporting reductions of 7.7–10.3% five minutes post-exercise, which remained evident up to 30 min post-intervention.

In contrast, eccentric exercise is known to induce EIMD/DOMS, leading to structural changes and inflammation in muscle and/or connective tissue [26] with inconclusive evidence on muscle stiffness. No significant negative consequences for muscle stiffness (the muscle gets stiffer) were reported after eccentric contractions in some studies [73, 74], while others showed a significant stiffness reduction after eccentric contraction (in the hamstrings).These findings align with our results [30] which calls for studies explaining variance of these effects. However, full ROM resistance training was effective in increasing acute flexibility [70, 75].

#### Device dependency

Whether the presented results align with previous literature crucially depends on the measurement device used to evaluate stiffness (causing a serious device objectivity problem with clinical relevance). While for the SWE measurement, stiffness significantly decreased after full ROM calf raises, myotonometry indicated an increase. The objectivity problem of the devices was outlined in previous studies [16], showing that the correlation between ROM and stiffness was dependent on SWE or myotonometry, as well. The authors hypothesized that measurement errors could be attributable to this construct validity limitation. Indeed, limited reliability was also found in previous literature on SWE (ICC for inter-operator and intra-operator reliability between 0.83 and 0.93) [71] and myotonometry (ICC = 0.64–0.98) [15]. Warneke et al. [16] highlighted the random absolute measurement errors that might contribute to the problem. However, in the present study, ICCs were between 0.621 and 0.990 and measurement errors ranged from 3.976% to 17.240%. Nevertheless, no systematic increase or decrease was reported in the control condition, questioning the exclusive attributability to measurement errors.

It seems that SWE and myotonometry measure two very different constructs [16]. SWE stiffness calculations assume a direct relationship between shear wave velocity and tissue stiffness and calculates the stiffness from the wave velocity of the tissue reflection. This method is considered as the most valid way to evaluate passive tissue stiffness in the literature [76], although there were some reliability concerns. The MyotonPRO quantifies stiffness via the response of the tissue to the mechanical impulse of 0.42 N, capturing oscillation frequency (Hz), dynamic stiffness (N/m), logarithmic decrement (representing tissue elasticity), mechanical stress relaxation time (ms), and creep. On the first view, the measurement seems more direct. However, (muscle) stiffness values may be biased by skin, fascia, subcutaneous fat and other connective tissue lying between the device and the muscle/tendon [34]. Although these limitations might have influenced the result, it is still surprising that myotonometry stiffness and SWE stiffness measurements point in opposite directions. A well-designed measurement device validation study should clarify these problems, as differences are of clinical relevance.

### Relevance of the results for ultrasound standardization

This study showed under controlled conditions that prior physical activities systematically influence critical ultrasound parameters (muscle thickness and stiffness). Since it cannot be assumed that all participants follow the same daily routines and have the same pre-measurement physical activity level, the results underscore the relevance of standardizing surrounding participant behavior for internal data validity of ultrasound measurements and minimize random errors. This includes controlling participants travel to the lab as well as the potential activities performed immediately before testing. Given evidence that DOMS-related oedema may persist up to 48 h [61–63], this calls for standardization of the physical activity up to 2 days before testing.

Furthermore, to ensure no enhanced activity-induced muscle swelling unsystematically biased ultrasound measurements, the results suggest important practical implications. In a best-case scenario, pre-measurement activity is standardized, avoiding extensive training routines with the potential for muscle swelling before testing. This also includes the transportation mode to arrive at the laboratory, which should be considered as minimal intensity. If such standardization is unrealistic or unpractical, the present results underscore the relevance of a sufficient resting time between arriving at the laboratory and starting the ultrasound measurement. Although depending on the pre-measurement activity, 10 min seem insufficient as a general recommendation since with intensive interventions (e.g., calf raises) the acute effects did not sufficiently dissipated. This statement is strengthened by previous literature in which retention to baseline took between 30 and 60 min [25]. This is of special interest as ultrasound measurements should be performed at the beginning of test-batteries, as maximum strength or other exhaustive routines with the potential for muscle swelling (inflammation) could bias ultrasound results meaningfully. If a standardization of pre-measurement participant arrival cannot be achieved, this could be used as a potential variable for variance explanation of ultrasound results and effort should be invested (e.g. via an exercise specific rest) to minimize pre-activity effects.

### Limitations

The three different active conditions produced muscle swelling of different effect sizes, indicating the largest effects for calf raises, smaller effects with jogging and no effects with cycling. These differences may reflect stimulus type, exercise intensity, or muscle involvement. Broader investigations across multiple muscle groups are needed to draw a more holistic and comprehensive picture of acute regional muscular adaptations. Furthermore, intensity between the different conditions is hard to standardize, as calf raises are a different stimulus compared to cycling or jogging. As in most other ultrasound studies, the pressure and probe angle could not be standardized in the ultrasound investigation. Examiner experience was the only possibility to optimize reliability. It cannot be ruled out that the training status of the participants caused specific effects as participants were accustomed to regular physical activity. Therefore, it cannot be guaranteed that the reported effects are observable in other populations (e.g., untrained or highly trained participants). Due to differences in exhaustion perception in different training levels (untrained versus highly trained) and the limitation that retention effect investigation was limited to 10 min after the post-test, the study results interpretation is limited to this time window, while further retention effects were necessary. In untrained populations, these interventions might be considered exhaustive and too fatiguing to be considered as just a warm-up. In this manner, the comparison of several populations with different activity histories (e.g., athletes vs. obese) concerning their musculotendinous parameters as well as their reaction to different exercise stimuli will ensure more accurate results and should be considered in future studies [33]. This is of special interest as numerous studies were performed without previous screening of pre-measurement activity and sitting times which might explain heterogeneity in stiffness results [32].

One further limitation can be seen in the surrounding conditions for the participants. The objective of the study was to investigate the acute effects of different exercise routines on muscle thickness and stiffness with a potential relevance on surrounding ultrasound standardization conditions. However, the transportation mode of participants was not standardized in this study. To counteract, a 10-minute rest between pre0 and pre10 was ensured and the influence of the rest on outcome parameters was systematically evaluated. However, it cannot be entirely ruled out that there was still a small influence. Another limitation refers to the retention effects. Previous studies indicated, depending on the stimulus and parameter, that a few minutes were insufficient and about 30 to 60 min may be necessary for muscle thickness to return to baseline values after an exhausting exercise task [25, 56]. However, this study provided a baseline to investigate whether a practical approach with 10 min resting counteracted enhanced physical activity induced structural changes in muscle thickness and stiffness. Future studies focusing on these effects should expand in more retention measurements to improve practical recommendations for future ultrasound standardization.

The MDC evaluation deserves special consideration when interpreting the results in terms of their clinical relevance [50, 51]. Systematic intervention-induced changes only surpassed the MDC in the calf raise condition for muscle thickness and MyotonPRO muscle stiffness evaluation, while the jogging condition showed statistically significant effects, while clinical relevance needs further investigation due to pre-post changes (e.g., muscle thickness 0.6 mm) were below the MDC (1.28 mm).

## Conclusion

Physical activity performed immediately before ultrasound testing has influence on measurement results. Not only resistance training, but also warm-up related activities (e.g., cycling or jogging) can impact sensitive muscle physical and mechanical property measurements. These activities that are common in everyday living call for standardization of pre-measurement physical activity (e.g., how participants arrive to the lab). Unstandardized surrounding conditions might explain comparatively high random measurement errors; therefore, future protocols should not focus exclusively on device-related parameters (probe pressure, angle, rotation, investigator and assessor experience). 

## Supplementary Information

Below is the link to the electronic supplementary material.

Supplementary Material 1

## Data Availability

Original data can be provided due to reasonable request from the corresponding author.
