# Supplementary material for: Should pre–measurement physical activity be standardized in muscle thickness and stiffness evaluations? – A randomized controlled four arm cross-over study
Source: BMC Med Imaging. 2026 Apr 29;26:285. doi: 10.1186/s12880-026-02373-5 (PMC13231601; doi:10.1186/s12880-026-02373-5)
Supplement: Supplementary file 1 — Supplementary Material 1 [file 12880_2026_2373_MOESM1_ESM.docx]

**Supplemental Material**

**Table S1** Test-retest reliability for myotonometry parameters within each test over the evaluation session

| **Parameter** | **ICC (CI95)** | **SEM** | **MDC** | **CV** | **MAE** | **MAPE** | **Bias** | **Bias_p** | **LoA_lower** | **LoA_upper** |
| --- | --- | --- | --- | --- | --- | --- | --- | --- | --- | --- |
| Creep pre0 | 0.977 (0.963-0.985) | 0.028 | 0.077 | 3.243 | 0.028 | 2.464 | 0.013 | 0.000 | -0.060 | 0.086 |
| Creep pre10 | 0.965 (0.946-0.977) | 0.035 | 0.098 | 4.085 | 0.036 | 3.416 | 0.015 | 0.001 | -0.079 | 0.109 |
| Creep post0 | 0.982 (0.966-0.989) | 0.029 | 0.080 | 3.406 | 0.030 | 2.721 | 0.016 | 0.000 | -0.057 | 0.090 |
| Creep post10 | 0.983 (0.975-0.989) | 0.025 | 0.070 | 3.138 | 0.026 | 2.377 | 0.008 | 0.017 | -0.061 | 0.077 |
| Creep AT pre0 | 0.891 (0.845-0.923) | 0.012 | 0.034 | 4.140 | 0.014 | 3.353 | 0.001 | 0.412 | -0.032 | 0.035 |
| Creep AT pre10 | 0.896 (0.852-0.927) | 0.013 | 0.037 | 4.499 | 0.013 | 3.139 | -0.001 | 0.583 | -0.038 | 0.036 |
| Creep AT post0 | 0.905 (0.866-0.934) | 0.014 | 0.038 | 4.627 | 0.013 | 3.193 | -0.002 | 0.334 | -0.040 | 0.036 |
| Creep AT post10 | 0.671 (0.555-0.761) | 0.024 | 0.066 | 8.043 | 0.017 | 4.033 | -0.004 | 0.265 | -0.070 | 0.063 |
| Decrement pre0 | 0.926 (0.894-0.948) | 0.042 | 0.117 | 5.864 | 0.044 | 4.355 | 0.002 | 0.765 | -0.116 | 0.119 |
| Decrement pre10 | 0.893 (0.849-0.925) | 0.049 | 0.135 | 6.712 | 0.053 | 5.092 | 0.001 | 0.881 | -0.135 | 0.137 |
| Decrement post0 | 0.922 (0.889-0.946) | 0.045 | 0.126 | 6.390 | 0.045 | 4.399 | 0.004 | 0.558 | -0.123 | 0.130 |
| Decrement post10 | 0.867 (0.812-0.907) | 0.059 | 0.163 | 8.150 | 0.052 | 5.396 | 0.016 | 0.040 | -0.145 | 0.178 |
| Decrement AT pre0 | 0.799 (0.721-0.857) | 0.080 | 0.221 | 13.375 | 0.086 | 10.008 | 0.005 | 0.654 | -0.218 | 0.227 |
| Decrement AT pre10 | 0.837 (0.771-0.885) | 0.079 | 0.219 | 13.268 | 0.079 | 9.287 | -0.005 | 0.669 | -0.225 | 0.216 |
| Decrement AT post0 | 0.864 (0.808-0.904) | 0.072 | 0.200 | 11.835 | 0.079 | 9.446 | 0.002 | 0.847 | -0.199 | 0.203 |
| Decrement AT post10 | 0.874 (0.822-0.912) | 0.067 | 0.185 | 11.139 | 0.073 | 8.704 | 0.011 | 0.205 | -0.173 | 0.196 |
| Frequency (Hz) pre0 | 0.965 (0.949-0.976) | 0.356 | 0.987 | 3.196 | 0.358 | 2.228 | -0.024 | 0.615 | -1.016 | 0.968 |
| Frequency (Hz) pre10 | 0.948 (0.922-0.965) | 0.432 | 1.198 | 3.802 | 0.422 | 2.723 | -0.165 | 0.004 | -1.328 | 0.997 |
| Frequency (Hz) post0 | 0.973 (0.961-0.981) | 0.395 | 1.095 | 3.425 | 0.383 | 2.352 | -0.081 | 0.125 | -1.172 | 1.009 |
| Frequency (Hz) post10 | 0.973 (0.961-0.981) | 0.360 | 0.999 | 3.195 | 0.357 | 2.227 | 0.036 | 0.462 | -0.967 | 1.039 |
| Frequency (Hz) AT pre0 | 0.813 (0.740-0.868) | 0.863 | 2.393 | 3.942 | 0.876 | 2.813 | -0.147 | 0.204 | -2.539 | 2.244 |
| Frequency (Hz) AT pre10 | 0.835 (0.769-0.883) | 0.875 | 2.425 | 4.028 | 0.921 | 2.953 | 0.085 | 0.471 | -2.350 | 2.520 |
| Frequency (Hz) AT post0 | 0.814 (0.741-0.868) | 0.952 | 2.640 | 4.386 | 1.006 | 3.276 | 0.174 | 0.173 | -2.461 | 2.809 |
| Frequency (Hz) AT post10 | 0.750 (0.656-0.821) | 0.990 | 2.744 | 4.596 | 0.996 | 3.246 | 0.042 | 0.753 | -2.716 | 2.800 |
| Relaxation (ms) pre0 | 0.979 (0.965-0.987) | 0.445 | 1.233 | 3.154 | 0.472 | 2.521 | 0.219 | 0.000 | -0.946 | 1.383 |
| Relaxation (ms) pre10 | 0.971 (0.946-0.983) | 0.527 | 1.460 | 3.557 | 0.579 | 3.081 | 0.306 | 0.000 | -1.036 | 1.648 |
| Relaxation (ms) post0 | 0.951 (0.922-0.968) | 0.826 | 2.290 | 6.178 | 0.579 | 3.578 | 0.379 | 0.000 | -1.806 | 2.563 |
| Relaxation (ms) post10 | 0.983 (0.974-0.989) | 0.437 | 1.213 | 3.244 | 0.463 | 2.522 | 0.173 | 0.003 | -0.999 | 1.346 |
| Relaxation (ms) AT pre0 | 0.914 (0.877-0.940) | 0.182 | 0.505 | 4.181 | 0.209 | 3.402 | 0.020 | 0.423 | -0.487 | 0.526 |
| Relaxation (ms) AT pre10 | 0.863 (0.807-0.904) | 0.270 | 0.748 | 6.121 | 0.217 | 3.384 | 0.004 | 0.902 | -0.748 | 0.757 |
| Relaxation (ms) AT post0 | 0.790 (0.709-0.851) | 0.358 | 0.991 | 8.135 | 0.249 | 4.171 | 0.012 | 0.810 | -0.985 | 1.008 |
| Relaxation (ms) AT post10 | 0.911 (0.873-0.938) | 0.199 | 0.551 | 4.527 | 0.209 | 3.335 | -0.009 | 0.739 | -0.564 | 0.546 |

Legende: ICC=Intraclass correlation coefficient, CI95=95% confidence interval, SEM=standard error of measurement, MDC=minimal detectable change, CV=variability coefficient, MAE=mean absolute error, MAPE=mean absolute percentage error, Bias=test-retest difference in measurement unit, Bias p=p-value of the paired sampled t-test, LoA=limits of agreement, AT=Achilles tendon

**Table S2** Interday test-retest reliability of myotonometry parameters for all testing conditions at the baseline and pre-test

| **Parameter** | **ICC (CI95)** | **SEM** | **MDC** | **CV** |
| --- | --- | --- | --- | --- |
| Creep pre0 | 0.962 (0.932-0.980) | 0.03 | 0.094785 | 8.38 |
| Creep pre10 | 0.969 (0.945-0.984) | 0.03 | 0.088978 | 7.74 |
| Creep post0 | 0.917 (0.667–0.970) | 0.05 | 0.151055 | 10.63 |
| Creep post10 | 0.950 (0.847–0.980) | 0.04 | 0.114227 | 8.39 |
| Creep AT pre0 | 0.829 (0.697-0.913) | 0.01 | 0.033558 | 8.23 |
| Creep AT pre10 | 0.821 (0.683-0.909) | 0.01 | 0.038231 | 9.32 |
| Creep AT post0 | 0.828 (0.684–0.914) | 0.01 | 0.040869 | 10.03 |
| Creep AT post10 | 0.814 (0.671–0.905) | 0.01 | 0.036626 | 8.97 |
| Decrement pre0 | 0.967 (0.942-0.983) | 0.03 | 0.073834 | 7.4 |
| Decrement pre10 | 0.962 (0.933-0.981) | 0.03 | 0.074906 | 7.4 |
| Decrement post0 | 0.913 (0.840–0.956) | 0.04 | 0.074906 | 11.28 |
| Decrement post10 | 0.948 (0.905–0.974) | 0.03 | 0.116982 | 9.07 |
| Decrement AT pre0 | 0.910 (0.840-0.954) | 0.05 | 0.126004 | 15.16 |
| Decrement AT pre10 | 0.869 (0.768-0.933) | 0.06 | 0.161031 | 19.29 |
| Decrement AT post0 | 0.832 (0.699–0.915) | 0.06 | 0.17593 | 20.91 |
| Decrement AT post10 | 0.934 (0.883–0.966) | 0.04 | 0.119995 | 14.43 |
| Frequency (Hz) pre0 | 0.955 (0.919-0.977) | 0.38 | 1.056904 | 6.83 |
| Frequency (Hz) pre10 | 0.963 (0.934-0.981) | 0.35 | 0.959639 | 6.29 |
| Frequency (Hz) post0 | 0.938 (0.799–0.976) | 0.55 | 1.52966 | 7.72 |
| Frequency (Hz) post10 | 0.953 (0.898–0.978) | 0.45 | 1.233582 | 6.83 |
| Frequency (Hz) AT pre0 | 0.837 (0.710-0.917) | 0.63 | 1.75796 | 5.84 |
| Frequency (Hz) AT pre10 | 0.782 (0.615-0.889) | 0.75 | 2.090433 | 6.88 |
| Frequency (Hz) AT post0 | 0.777 (0.601–0.887) | 0.77 | 2.135893 | 7.19 |
| Frequency (Hz) AT post10 | 0.784 (0.616–0.890) | 0.68 | 1.872569 | 6.26 |
| Relaxation (ms) pre0 | 0.957 (0.924-0.978) | 0.6 | 1.671041 | 9.01 |
| Relaxation (ms) pre10 | 0.971 (0.948-0.985) | 0.51 | 1.417374 | 7.53 |
| Relaxation (ms) post0 | 0.910 (0.644–0.968) | 0.98 | 2.727161 | 11.81 |
| Relaxation (ms) post10 | 0.951 (0.845–0.980) | 0.7 | 1.937165 | 8.61 |
| Relaxation (ms) AT pre0 | 0.850 (0.734-0.923) | 0.2 | 0.54704 | 8.99 |
| Relaxation (ms) AT pre10 | 0.781 (0.611-0.888) | 0.26 | 0.715455 | 11.68 |
| Relaxation (ms) AT post0 | 0.803 (0.641–0.901) | 0.26 | 0.722212 | 11.99 |
| Relaxation (ms) AT post10 | 0.852 (0.737–0.924) | 0.21 | 0.582833 | 9.56 |

Legend: ICC=Intraclass correlation coefficient, CI95=95% confidence interval, SEM=standard error of measurement, MDC=minimal detectable change, CV=variability coefficient, AT=Achilles tendon

**Table S3** shows test descriptives separated for the intervention conditions with the following two-way ANOVA results

|  | **Condition** | **Initial test** | **Pretest** | **Posttest** | **Retention test** | **Main Effect Condition** | **Main Effect Time** | **Time** $\boldsymbol{\times}$ **Condition Interaction** |
| --- | --- | --- | --- | --- | --- | --- | --- | --- |
| MyotonPRO Frequency  (Hz)  AT | Calf Raise | 31.34±1.99 | 31.40±2.03 | 31.58±2.27 | 30.97±2.19 | F(2.47, 66.74) = 3.61  p = 0.024  ηp² = 0.118 | F(2.28, 61.56) = 2.19  p = 0.114  ηp² = 0.075 | F(5.73, 154.79) = 1.25  p = 0.287  ηp² = 0.044 |
|  | Jogging | 30.50±2.05 | 30.80±2.33 | 30.08±2.18 | 30.47±2.10 |  |  |  |
|  | Cycling | 30.81±1.72 | 30.43±1.70 | 30.05±2.56 | 30.47±1.71 |  |  |  |
|  | Control | 31.44±1.82 | 30.96±2.10 | 31.04±1.90 | 30.94±1.58 |  |  |  |
| MyotonPRO Stiffness  (N/m) AT | Calf Raise | 817.53±80.00 | 814.43±77.78 | 839.08±79.69 | 825.57±79.58 | F(2.40, 64.80) = 3.39  p = 0.032  ηp² = 0.111 | F(2.58, 69.74) = 0.78  p = 0.491  ηp² = 0.028 | F(5.71, 154.06) = 1.19  p = 0.315  ηp² = 0.042 |
|  | Jogging | 804.29±76.35 | 807.21±85.72 | 790.16±89.15 | 797.00±89.81 |  |  |  |
|  | Cycling | 805.12±84.13 | 792.41±75.65 | 788.60±95.26 | 792.88±99.20 |  |  |  |
|  | Control | 833.15±73.17 | 819.55±82.46 | 819.92±82.59 | 813.73±62.88 |  |  |  |
| MyotonPRO Decrement AT | Calf Raise | 0.84±0.15 | 0.82±0.16 | 0.82±0.15 | 0.83±0.18 | F(2.01, 54.19) = 2.50  p = 0.091  ηp² = 0.085 | F(2.17, 58.73) = 1.05  p = 0.360  ηp² = 0.038 | F(6.11, 165.00) = 1.76  p = 0.108  ηp² = 0.061 |
|  | Jogging | 0.85±0.17 | 0.82±0.17 | 0.87±0.18 | 0.17±0.17 |  |  |  |
|  | Cycling | 0.85±0.18 | 0.89±0.22 | 0.92±0.21 | 0.86±0.20 |  |  |  |
|  | Control | 0.83±0.17 | 0.84±0.19 | 0.83±0.20 | 0.84±0.18 |  |  |  |
| MyotonPRO  Relaxation  (ms) AT | Calf Raise | 6.17±0.65 | 6.18±0.66 | 5.98±0.62 | 6.09±0.59 | F(2.43, 65.53) = 4.05  p = 0.016  ηp² = 0.130 | F(2.46, 66.34) = 1.53  p = 0.220  ηp² = 0.054 | F(5.72, 154.55) = 1.43  p = 0.208  ηp² = 0.050 |
|  | Jogging | 6.26±0.67 | 6.33±0.84 | 6.41±0.78 | 6.36±0.81 |  |  |  |
|  | Cycling | 6.20±0.55 | 6.40±0.64 | 6.45±0.82 | 6.32±0.68 |  |  |  |
|  | Control | 6.03±0.54 | 5.98±0.62 | 6.07±0.64 | 6.16±0.47 |  |  |  |
| MyotonPRO  Creep  AT | Calf Raise | 0.41±0.04 | 0.41±0.04 | 0.40±0.04 | 0.41±0.04 | F(2.47, 66.66) = 4.17  p = 0.014  ηp² = 0.134 | F(2.27, 61.32) = 1.10  p = 0.346  ηp² = 0.039 | F(5.90, 159.17) = 1.61  p = 0.149  ηp² = 0.056 |
|  | Jogging | 0.42±0.04 | 0.42±0.04 | 0.43±0.05 | 0.43±0.05 |  |  |  |
|  | Cycling | 0.42±0.03 | 0.43±0.04 | 0.43±0.05 | 0.42±0.04 |  |  |  |
|  | Control | 0.41±0.03 | 0.41±0.04 | 0.41±0.04 | 0.41±0.04 |  |  |  |

Legend: AT=Achilles tendon, F=F-statistic, p=p-value, ηp²=partial eta squared

**Table S4** Test descriptives separated for the intervention conditions with the following two-way ANOVA results for muscle parameter

|  | **Condition** | **Pre0** | **Pre10** | **Post0** | **Post10** | **Main Effect Condition** | **Main Effect**  **Time** | **Time** $\boldsymbol{\times}$ **Condition Interaction** |
| --- | --- | --- | --- | --- | --- | --- | --- | --- |
| MyotonPRO  Frequeny  (Hz) | Calf Raise | 16.06±2.06 | 15.72±1.84 | 17.63±2.91 | 17.00±2.88 | F(2.53, 68.20) = 14.69  p = <.001  ηp² = 0.352 | F(2.04, 55.16) = 13.21  p = <.001  ηp² = 0.329 | F(4.26, 114.94) = 11.27  p = <.001  ηp² = 0.294 |
|  | Jogging | 15.88±1.98 | 15.65±1.95 | 16.25±2.23 | 16.08±2.05 |  |  |  |
|  | Cycling | 15.75±1.75 | 15.64±1.68 | 15.66±1.82 | 15.82±1.94 |  |  |  |
|  | Control | 16.11±2.07 | 15.77±2.20 | 15.86±2.17 | 15.69±1.92 |  |  |  |
| MyotonPRO  Decrement | Calf Raise | 1.03±0.15 | 1.04±0.14 | 0.97±0.14 | 1.00±0.17 | F(2.90, 78.41) = 4.72  p = 0.005  ηp² = 0.149 | F(2.74, 73.87) = 6.39  p = <.001  ηp² = 0.191 | F(3.91, 105.46) = 2.55  p = 0.045  ηp² = 0.086 |
|  | Jogging | 1.02±0.17 | 1.02±0.15 | 1.00±0.13 | 1.00±0.16 |  |  |  |
|  | Cycling | 1.01±0.13 | 1.03±0.14 | 1.04±0.20 | 1.04±0.17 |  |  |  |
|  | Control | 1.03±0.15 | 1.04±0.15 | 1.03±0.14 | 1.03±0.14 |  |  |  |
| MyotonPRO  Relaxation  (ms) | Calf Raise | 18.74±3.19 | 19.17±3.22 | 15.31±4.05 | 16.73±3.70 | F(2.61, 70.39) = 19.28  p = <.001  ηp² = 0.417 | F(1.91, 51.60) = 25.78  p = <.001  ηp² = 0.488 | F(4.10, 110.81) = 29.31  p = <.001  ηp² = 0.520 |
|  | Jogging | 18.83±3.16 | 19.08±3.06 | 18.18±3.31 | 18.42±3.17 |  |  |  |
|  | Cycling | 18.94±2.74 | 19.33±2.75 | 19.16±2.96 | 19.10±3.02 |  |  |  |
|  | Control | 18.36±3.27 | 18.97±3.38 | 18.94±3.28 | 19.02±3.06 |  |  |  |
| MyotonPRO  Creep | Calf Raise | 1.14±0.19 | 1.17±0.16 | 0.95±0.23 | 1.02±0.22 | F(2.65, 71.44) = 18.07  p = <.001  ηp² = 0.401 | F(1.95, 52.65) = 27.88  p = <.001  ηp² = 0.508 | F(3.99, 107.65) = 26.17  p = <.001  ηp² = 0.492 |
|  | Jogging | 1.15±0.19 | 1.17±0.18 | 1.11±0.19 | 1.12±0.19 |  |  |  |
|  | Cycling | 1.15±0.17 | 1.18±0.16 | 1.16±0.17 | 1.16±0.18 |  |  |  |
|  | Control | 1.12±0.19 | 1.15±0.21 | 1.15±0.19 | 1.16±0.18 |  |  |  |

Legend: F=F-statistic, p=p-value, ηp²=partial eta squared

**Table S5** Significant within-condition change from baseline test (pre0) to Pretest (pre10). Entries are paired-samples mean differences pre0-pre10 per condition (negative values = higher values at pre10 than pre0; positive = lower at pre10).

| **Parameter** | **Condition** | **Time** | **Estimate** | **CI95** | **SE** | **t** | **df** | **p-value** | **Cohens d** |
| --- | --- | --- | --- | --- | --- | --- | --- | --- | --- |
| MyotonPRO Relaxation (ms) | Control | pre0 - pre10 | -0.659 | [-1.078, -0.240] | 0.147 | -4.474 | 27 | 0.001 | -0.845 |
| MyotonPRO Creep | Calf raises | pre0 - pre10 | -0.026 | [-0.053, 0.001] | 0.009 | -2.768 | 27 | 0.010 | -0.523 |
| MyotonPRO Relaxation (ms) | Calf raises | pre0 - pre10 | -0.382 | [-0.838, 0.074] | 0.160 | -2.385 | 27 | 0.024 | -0.451 |
| MyotonPRO Creep | Control | pre0 - pre10 | -0.033 | [-0.063, -0.002] | 0.011 | -2.998 | 27 | 0.027 | -0.567 |
| MyotonPRO Frequency (Hz) | Calf raises | pre0 - pre10 | 0.291 | [-0.107, 0.689] | 0.140 | 2.080 | 27 | 0.047 | 0.393 |

Legende: CI95=95% confidence interval, SE=standard error, C=creep, D=density, F=frequency, R=relaxation, S= stiffness, t=t-value, df= degrees of freedom

At the Pre10, the control condition was significantly higher at pre10 for relaxation (difference=-0.659 ms (-3.4%), p=0.001, d=-0.845) compared to Pre0. The MDC here was 1.16–1.34 ms. Further effects were observed in the myotonometry test for muscle stiffness being significantly lower (difference=9.464 Nm (3.4%), p=0.002, d=0.766, MDC=28.32-50.66 Nm), creep was higher (difference=-0.033 (-2.8%), p=0.027, d=-0.567, MDC=2.29–2.89) in the pre10 compared to pre0. In the calf raise condition, the creep and relaxation parameter showed a significant increase from pre0 to pre10 (difference=-0.026 (-2.2%), p=0.010, d=-0.523, MDC=2.29–2.89; difference=-0.382 (-1.9%), p=0.024, d=-0.451, MDC=1.16–1.34 ms), while frequency decreased (difference=0.291 Hz (1,9%), p=0.047, d=0.393, MDC=0.99–1.16 Hz). Between conditions between day comparisons showed no significant differences, indicating similar baseline values (p=0.179–1.00).

**Table S6** Significant within-condition changes between the Pre-Test, Post-Test and Retention-Test. For each parameter and condition, entries are paired-samples mean differences for Pre-Post (pre10-post0), Pre-Retention (pre10-post10), and Post-Retention (post0-post10).

| **Parameter** | **Condition** | **Time** | **Estimate** | **CI95** | **SE** | **t** | **df** | **p-value** | **Cohens d** |
| --- | --- | --- | --- | --- | --- | --- | --- | --- | --- |
| MyotonPRO Creep | Calf raises | pre10 - post0 | 0.215 | [0.148, 0.283] | 0.024 | 9.062 | 27 | <0.001 | 1.713 |
|  | Calf raises | pre10 - post10 | 0.146 | [0.076, 0.216] | 0.024 | 5.977 | 27 | <0.001 | 1.129 |
|  | Calf raises | post0 - post10 | -0.069 | [-0.106, -0.033] | 0.013 | -5.397 | 27 | <0.001 | -1.020 |
|  | Jogging | pre10 - post0 | 0.056 | [0.017, 0.096] | 0.014 | 4.094 | 27 | 0.002 | 0.774 |
|  | Jogging | pre10 - post10 | 0.043 | [0.008, 0.077] | 0.012 | 3.484 | 27 | 0.009 | 0.658 |
| MyotonPRO Relaxation (ms) | Calf raises | pre10 - post0 | 3.766 | [2.580, 4.953] | 0.417 | 9.036 | 27 | <0.001 | 1.708 |
|  | Calf raises | pre10 - post10 | 2.398 | [1.227, 3.569] | 0.411 | 5.832 | 27 | <0.001 | 1.102 |
|  | Calf raises | pre0 - post10 | -1.368 | [-2.030, -0.706] | 0.234 | -5.881 | 27 | <0.001 | -1.111 |
|  | Jogging | pre10 - post0 | 0.894 | [0.231, 1.554] | 0.232 | 3.842 | 27 | 0.004 | 0.726 |
|  | Jogging | pre10 - post10 | 0.652 | [0.063, 1.240] | 0.207 | 3.153 | 27 | 0.020 | 0.596 |
| MyotonPRO Frequency (Hz) | Calf raises | pre10 - post0 | -1.879 | [-2.748, -1.009] | 0.305 | -6.152 | 27 | <0.001 | -1.163 |
|  | Calf raises | post0 - post10 | 0.736 | [0.325, 1.146] | 0.144 | 5.101 | 27 | <0.001 | 0.964 |
|  | Calf raises | pre10 - post10 | -1.143 | [-1.903, -0.382] | 0.267 | -4.278 | 27 | 0.001 | -0.808 |
|  | Jogging | pre10 - post0 | -0.596 | [-1.053, -0.140] | 0.160 | -3.722 | 27 | 0,006 | -0.703 |
| MyotonPRO Decrement | Calf raises | pre10 - post0 | 0.071 | [0.022, 0.120] | 0.017 | 4.144 | 27 | 0.002 | 0.783 |

Legende: CI95=95% confidence interval, SE= standard error, SWE=shear wave elastography, AT=Achilles tendon, t=t-value, df=degrees of freedom

- - 1. *Additional parameters*

For calf raises, creep and relaxation measured with myotonometry decreased from pre10 to post0 (creep: difference=0.215 (22.3%), p<0.001, d=1.713; relaxation: difference=3.766 ms (24.1%), p<0.001, d=1.708) and from pre10 to post10 (creep: difference=0.146 (14.1%), p<0.001, d=1.129; relaxation: difference=2.398 ms (14.1%), p<0.001, d=1.102), with partial rebounds from post0 to post10 (creep: difference=-0.069 (-6.7%), p<0.001, d=-1.020; relaxation: difference=-1.368 ms (13.6%), p<0.001, d=-1.111). Frequency increased from pre10 to post0 (difference=-1.879 Hz (-10.1%), p<0.001, d=-1.163), partially regressed from post0 to post10 (difference=0.736 Hz (4.4%), p<0.001, d=0.964), and remained elevated (difference=-1.143 Hz (-6.8%), p=0.001, d=-0.808). Decrement increased significantly after calf raises (difference: 0.071 (7.42%), p=0.002, d=0.783). For jogging frequency increased pre10 to post0 (difference=-0.596 Hz (-3.7%), p=0.006, d=-0.703), while creep and relaxation decreased from pre10 to post0 and post10 (creep: difference=0.056 (5.1%), p=0.002, d=0.774; difference=0.043 (3.8%), p=0.009, d=0.658; relaxation: difference=0.894 ms (4.9%), p=0.004, d=0.726; difference=0.652 ms (3.5%), p=0.020, d=0.596, see Figure S5-S8).

**Table S7** Significant pairwise between-condition contrasts of the within-subject change from Pretest (pre10) to Posttest (post0). Entries show the estimated mean difference of the change (pre10-post0) between the two conditions (“first - second”). Negative values therefore indicate a larger increase from pre10 to post0 in the first condition compared with the second.

| **Parameter** | **Time** | **Condition** | **Rise** | **CI95** | **SE** | **t** | **df** | **p-value** | **Cohens d** |
| --- | --- | --- | --- | --- | --- | --- | --- | --- | --- |
| MyotonPRO Creep | pre10 - post0 | Calf raises - Bike | 0.202 | [0.115, 0.288] | 0.024 | 8.332 | 27 | <0.001 | 1.575 |
|  | pre10 - post0 | Calf raises - Control | 0.217 | [0.125, 0.308] | 0.026 | 8.418 | 27 | <0.001 | 1.591 |
|  | pre10 - post0 | Calf raises - Jogging | 0.159 | [0.095, 0.222] | 0.018 | 8.889 | 27 | <0.001 | 1.680 |
|  | pre10 - post0 | Jogging - Control | 0.058 | [0.003, 0.112] | 0.015 | 3.791 | 27 | 0.017 | 0.716 |
| MyotonPRO Frequency (Hz) | pre10 - post0 | Calf raises - Bike | -1.877 | [-2.981, -0.773] | 0.310 | -6.059 | 27 | <0.001 | -1.145 |
|  | pre10 - post0 | Calf raises - Control | -1.773 | [-2.883, -0.663] | 0.312 | -5.691 | 27 | <0.001 | -1.075 |
|  | pre10 - post0 | Calf raises - Jogging | -1.282 | [-2.204, -0.360] | 0.259 | -4.955 | 27 | 0.001 | -0.936 |
|  | pre10 - post0 | Jogging - Bike | -0.595 | [-1.136, -0.054] | 0.152 | -3.917 | 27 | 0.016 | -0.740 |
| MyotonPRO Relaxation (ms) | pre10 - post0 | Calf raises - Bike | 3.636 | [2.054, 5.217] | 0.444 | 8.192 | 27 | <0.001 | 1.548 |
|  | pre10 - post0 | Calf raises - Control | 3.732 | [2.263, 5.201] | 0.412 | 9.052 | 27 | <0.001 | 1.711 |
|  | pre10 - post0 | Calf raises - Jogging | 2.873 | [1.645, 4.102] | 0.345 | 8.333 | 27 | <0.001 | 1.575 |
|  | pre10 - post0 | Jogging - Control | 0.859 | [-0.012, 1.730] | 0.244 | 3.515 | 27 | 0.031 | 0.664 |

Legend: SWE=shear wave elastography, CI95=95% confidence interval, SE=standard error, t=t-value, df=degrees of freedom

*Additional parameters (myotonometry)*

Further significant post hoc tests indicated decreasing creep more after calf raises than after cycling, control or jogging (difference=0.159-0.217, all p<.001, d=1.575-1.680) with 12.8-17.3% difference between conditions in post0 and jogging versus control (difference=0.058, p=0.017, d=0.716) with 4.6% difference in post0. Frequency increased more after calf raises than after cycling, control or jogging (difference=-1.877 to -1.282, all p≤0.001, d=-1.145 to -0.936) with 7.4 to 11.8% difference between condition in post0, and jogging exceeded cycling (difference=-0.595, p=0.016, d=-0.740) with 4.1% difference in post0. The relaxation parameter measured via myotonometry showed significant decreases after calf raises than after the remaining conditions (difference=2.873-3.732, all p<.001, d=1.548-1.711) with -14.2--19.1% difference between conditions in post0. Jogging also decreasing more than control (difference=0.859, p=0.031, d=0.664) with 4.8% in post0. Table 4 synthesizes significant effects, while all post-hoc results are reported in excel table in the **Supplemental Material**.

**Table S8** Significant retention (post10) between-condition comparisons. Entries are paired within-subject mean differences at post10 for each parameter and condition contrasts

| **Parameter** | **Time** | **Condition** | **Estimate** | **CI95** | **SE** | **t** | **df** | **p-value** | **Cohens d** |
| --- | --- | --- | --- | --- | --- | --- | --- | --- | --- |
| Muscle thickness (mm) | post10 | Calf raises - Control | 0.973 | [0.175, 1.770] | 0.280 | 3.471 | 27 | 0.009 | 0.656 |
|  | post10 | Jogging - Control | 0.724 | [0.160, 1.288] | 0.198 | 3.655 | 27 | 0.007 | 0.691 |
| MyotonPROCreep | post10 | Calf raises - Bike | -0.128 | [-0.171, -0.085] | 0.015 | -8.407 | 27 | <0.001 | -1.589 |
|  | post10 | Calf raises - Control | -0.129 | [-0.184, -0.075] | 0.019 | -6.739 | 27 | <0.001 | -1.274 |
|  | post10 | Calf raises - Jogging | -0.086 | [-0.136, -0.036] | 0.018 | -4.862 | 27 | <0.001 | -0.919 |
|  | post10 | Jogging - Bike | -0.042 | [-0.077, -0.007] | 0.012 | -3.393 | 27 | 0.006 | -0.641 |
|  | post10 | Jogging - Control | -0.043 | [-0.082, -0.005] | 0.014 | -3.196 | 27 | 0.007 | -0.604 |
| MyotonPRO Frequency (Hz) | post10 | Calf raises - Bike | 0.939 | [0.318, 1.561] | 0.218 | 4.303 | 27 | 0.001 | 0.813 |
|  | post10 | Calf raises - Control | 1.188 | [0.435, 1.940] | 0.264 | 4.490 | 27 | 0.001 | 0.849 |
|  | post10 | Calf raises - Jogging | 0.623 | [-0.039, 1.285] | 0.233 | 2.680 | 27 | 0.037 | 0.506 |
|  | post10 | Jogging - Bike | 0.316 | [-0.030, 0.662] | 0.121 | 2.603 | 27 | 0.037 | 0.492 |
|  | post10 | Jogging - Control | 0.564 | [0.060, 1.069] | 0.177 | 3.183 | 27 | 0.015 | 0.601 |
| MyotonPRO Relaxation (ms) | post10 | Calf raises - Bike | -2.229 | [-2.942, -1.515] | 0.251 | -8.893 | 27 | <0.001 | -1.681 |
|  | post10 | Calf raises - Control | -2.198 | [-3.121, -1.276] | 0.324 | -6.782 | 27 | <0.001 | -1.282 |
|  | post10 | Calf raises - Jogging | -1.455 | [-2.309, -0.602] | 0.300 | -4.854 | 27 | <0.001 | -0.917 |
|  | post10 | Jogging - Bike | -0.773 | [-1.336, -0.210] | 0.198 | -3.908 | 27 | 0.002 | -0.739 |
|  | post10 | Jogging - Control | -0.743 | [-1.389, -0.097] | 0.227 | -3.272 | 27 | 0.006 | -0.618 |
| MyotonPRO Stiffness (N/m) | post10 | Calf raises - Bike | 31.018 | [0.864, 61.171] | 10.592 | 2.928 | 27 | 0.027 | 0.553 |
|  | post10 | Calf raises - Control | 36.161 | [14.620, 57.702] | 7.567 | 4.779 | 27 | <0.001 | 0.903 |
|  | post10 | Calf raises - Jogging | 25.661 | [6.516, 44.806] | 6.725 | 3.816 | 27 | 0.004 | 0.721 |
|  | post10 | Jogging - Control | 10.500 | [-0.524, 21.524] | 3.872 | 2.712 | 27 | 0.035 | 0.512 |
| SWE Muscle Stiffness (kPa) | post10 | Calf raises - Bike | -1.643 | [-3.311, 0.025] | 0.586 | -2.804 | 27 | 0.037 | -0.53 |
|  | post10 | Calf raises - Control | -2.441 | [-3.640, -1.242] | 0.421 | -5.795 | 27 | <0.001 | -1.095 |
|  | post10 | Calf raises - Jogging | -1.480 | [-2.790, -0.171] | 0.460 | -3.218 | 27 | 0.017 | -0.608 |

Legend: SWE=shear wave elastography, CI95=95% confidence interval, SE=standard error, t=t-value, df=degrees of freedom

Figure list:


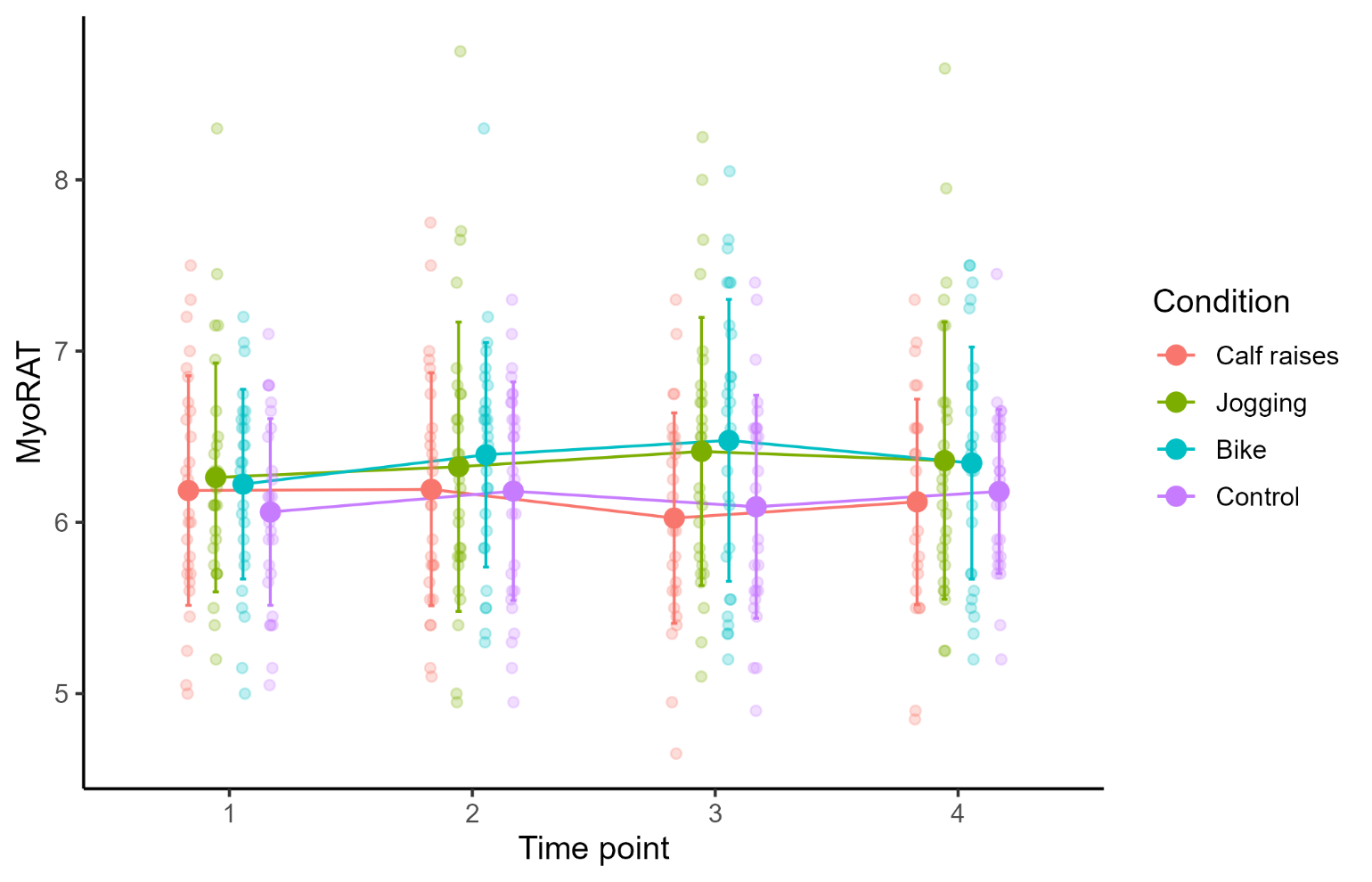


**Figure S1** Mean and standard deviation of the Achilles tendon relaxation measured by myotonometry at different time points for all conditions. MyoRAT = Achilles tendon relaxation, Time point 1 = pre0 testing before 10 minutes of rest, Time point 2 = pre10 testing serving as the baseline value for the different interventions, Time point 3= post0 testing as the post intervention test and Time point 4 = post10 reflecting the 10 minutes retention test


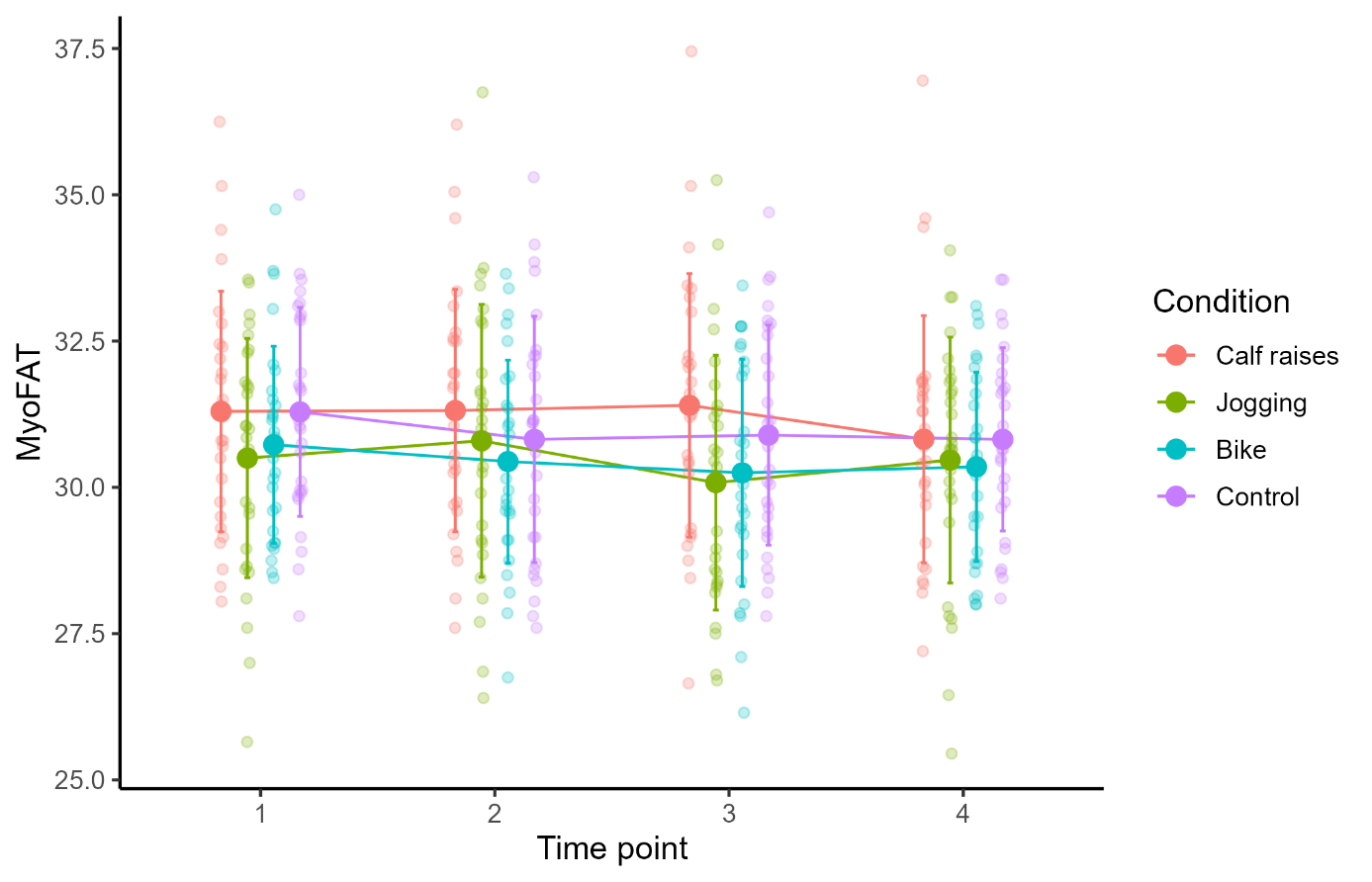


**Figure S2** Mean and standard deviation of the Achilles tendon frequency measured by myotonometry at different time points for all conditions. MyoFAT = Achilles tendon frequency, Time point 1 = pre0 testing before 10 minutes of rest, Time point 2 = pre10 testing serving as the baseline value for the different interventions, Time point 3= post0 testing as the post intervention test and Time point 4 = post10 reflecting the 10 minutes retention test


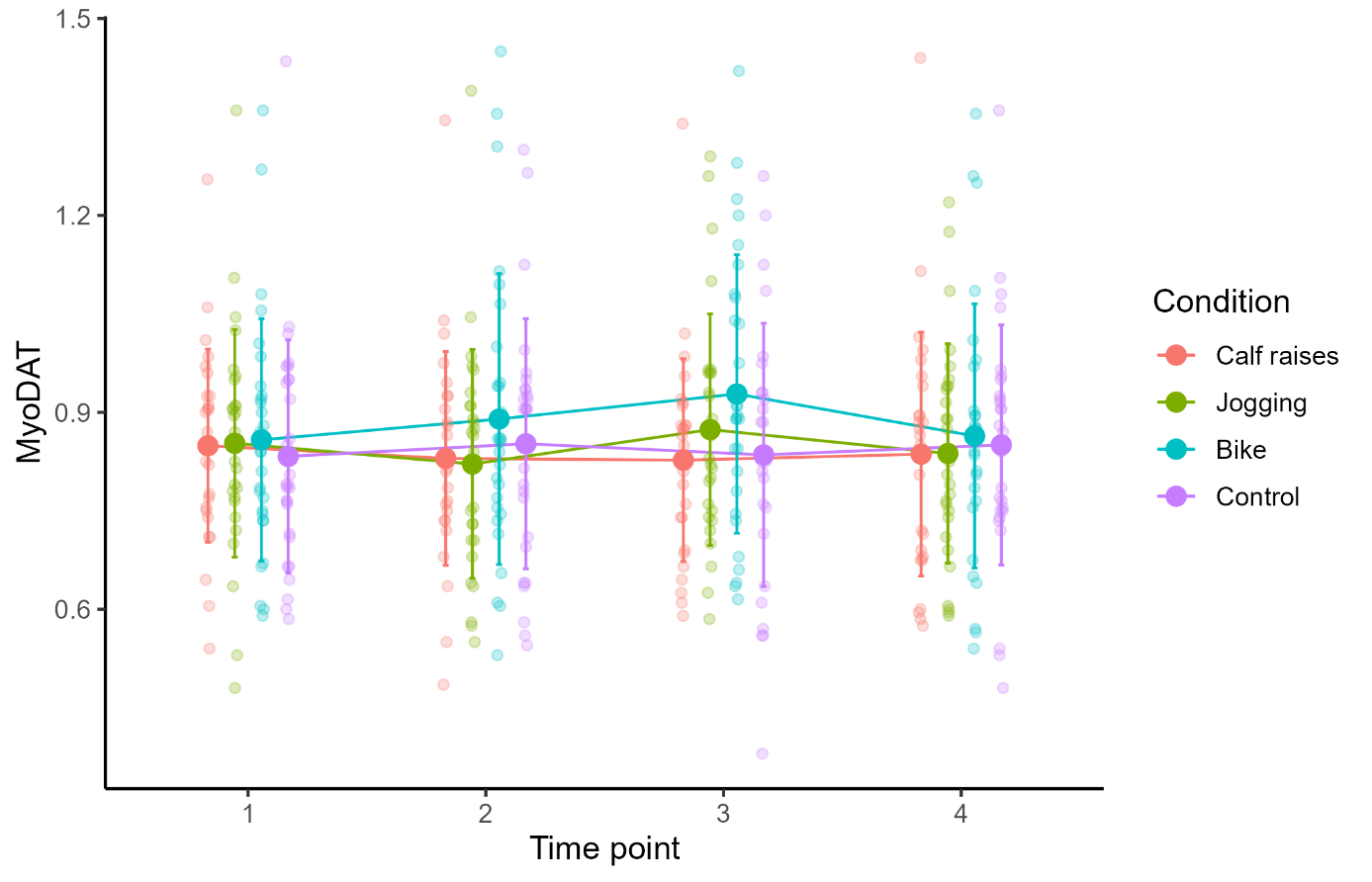


**Figure S3** Mean and standard deviation of the Achilles tendon decrement measured by myotonometry at different time points for all conditions. MyoDAT = Achilles tendon decrement, Time point 1 = pre0 testing before 10 minutes of rest, Time point 2 = pre10 testing serving as the baseline value for the different interventions, Time point 3= post0 testing as the post intervention test and Time point 4 = post10 reflecting the 10 minutes retention test


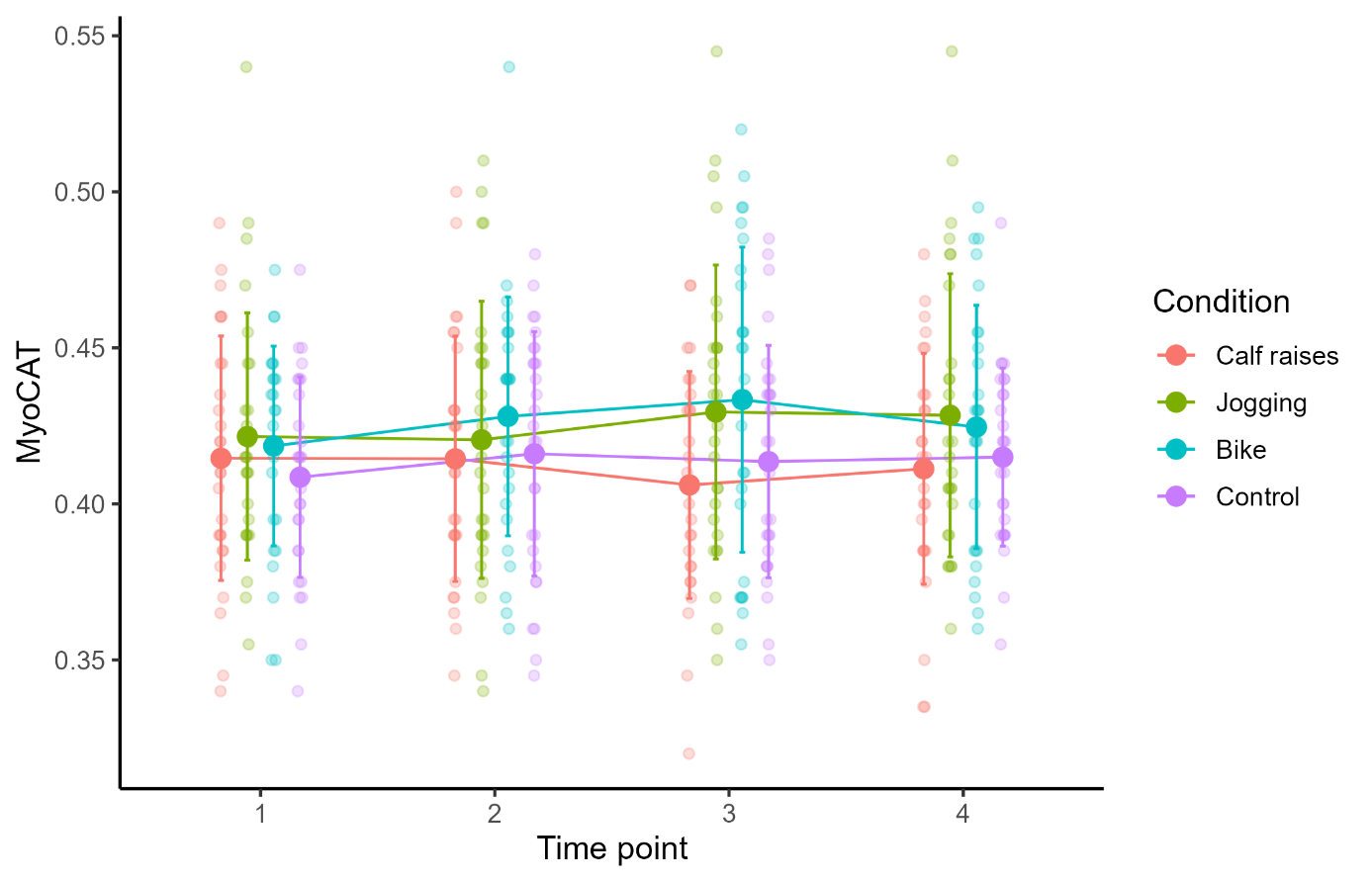


**Figure S4** Mean and standard deviation of the Achilles tendon creep measured by myotonometry at different time points for all conditions. MyoCAT = Achilles tendon creep, Time point 1 = pre0 testing before 10 minutes of rest, Time point 2 = pre10 testing serving as the baseline value for the different interventions, Time point 3= post0 testing as the post intervention test and Time point 4 = post10 reflecting the 10 minutes retention test


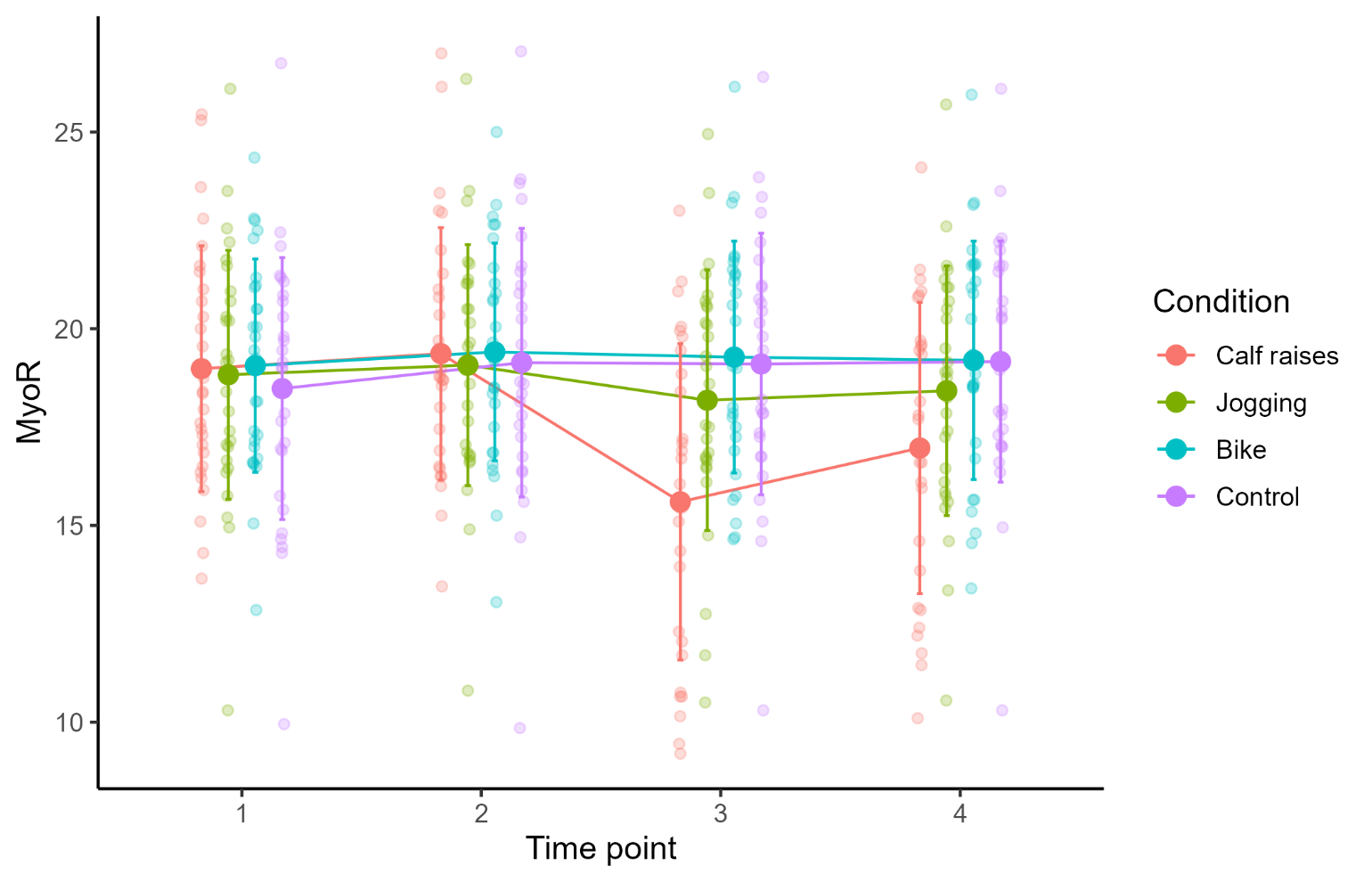


**Figure S5** Mean and standard deviation of the muscle relaxation measured by myotonometry at different time points for all conditions. MyoR = muscle relaxation, Time point 1 = pre0 testing before 10 minutes of rest, Time point 2 = pre10 testing serving as the baseline value for the different interventions, Time point 3= post0 testing as the post intervention test and Time point 4 = post10 reflecting the 10 minutes retention test


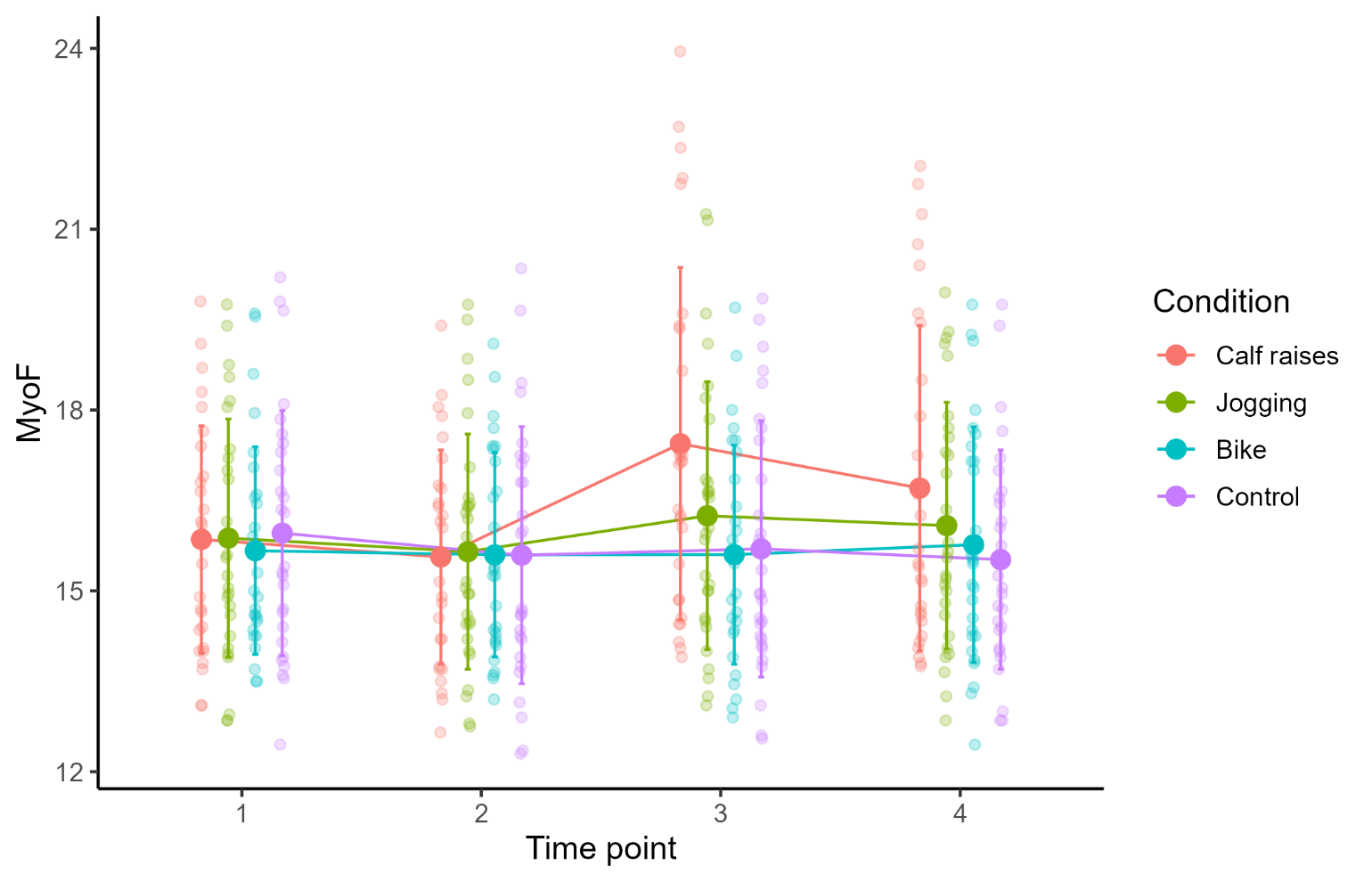


**Figure S6** Mean and standard deviation of the muscle frequency measured by myotonometry at different time points for all conditions. MyoF = muscle frequency, Time point 1 = pre0 testing before 10 minutes of rest, Time point 2 = pre10 testing serving as the baseline value for the different interventions, Time point 3= post0 testing as the post intervention test and Time point 4 = post10 reflecting the 10 minutes retention test


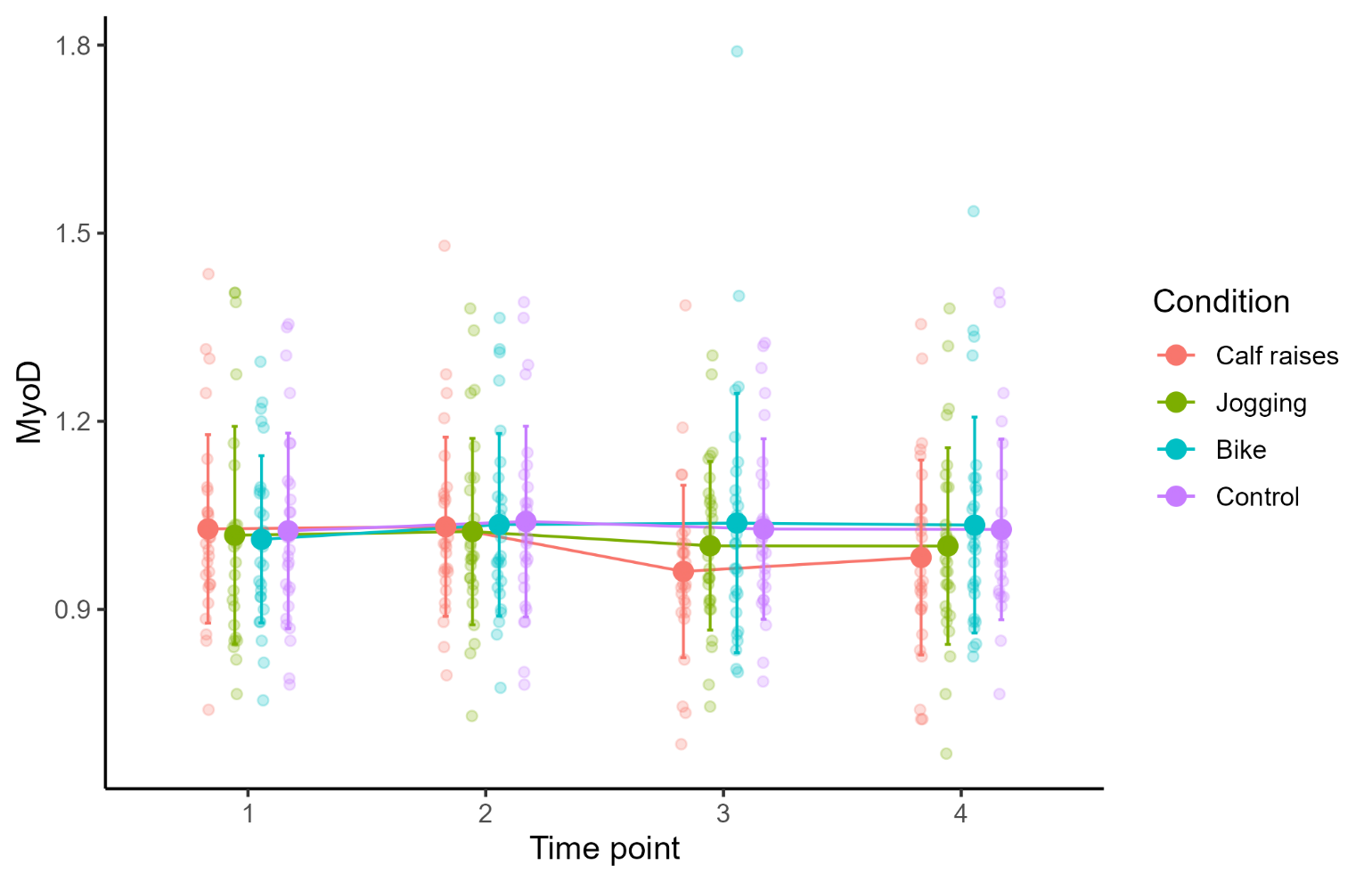


**Figure S7** Mean and standard deviation of the muscle decrement measured by myotonometry at different time points for all conditions. MyoD = muscle decrement, Time point 1 = pre0 testing before 10 minutes of rest, Time point 2 = pre10 testing serving as the baseline value for the different interventions, Time point 3= post0 testing as the post intervention test and Time point 4 = post10 reflecting the 10 minutes retention test


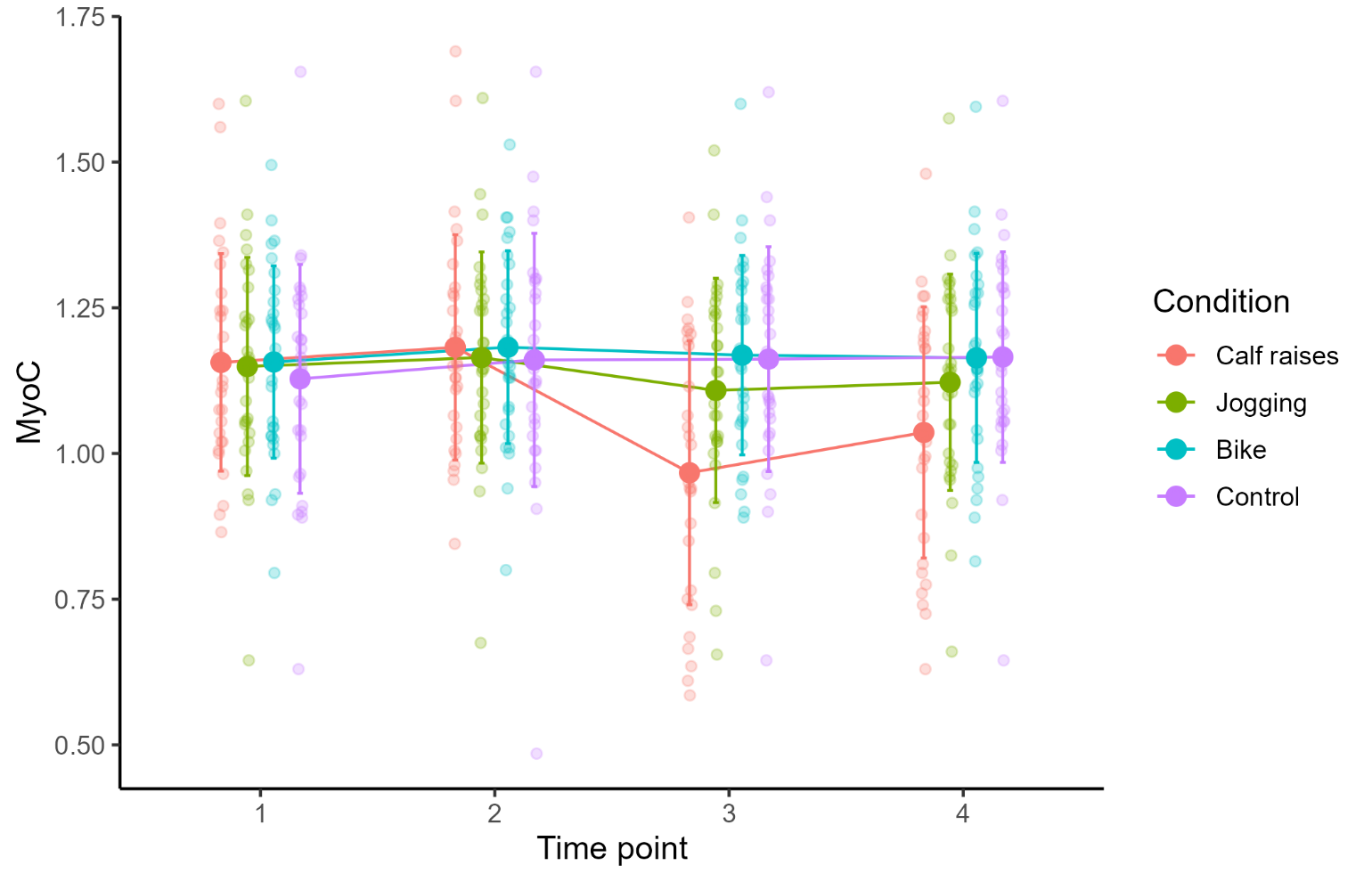


**Figure S8** Mean and standard deviation of the muscle creep measured by myotonometry at different time points for all conditions, MyoC = muscle creep, Time point 1 = pre0 testing before 10 minutes of rest, Time point 2 = pre10 testing serving as the baseline value for the different interventions, Time point 3= post0 testing as the post intervention test and Time point 4 = post10 reflecting the 10 minutes retention test
